# Ethnicity, information and cooperation: Evidence from a group-based nutrition intervention^☆^

**DOI:** 10.1016/j.foodpol.2023.102478

**Published:** 2023-10

**Authors:** Kalyani Raghunathan, Muzna Alvi, Mrignyani Sehgal

**Affiliations:** a International Food Policy Research Institute, New Delhi, India; b Department of Agricultural and Consumer Economics, University of Illinois Urbana-Champaign, United States

**Keywords:** Ethnicity, Self-help groups, India, Kitchen gardens, Behavior change communication, Field experiment

## Abstract

Development programs often rely on locally hired agents for service delivery, especially for interventions promoting agricultural practices, health, and nutrition. These agents are key to reaching underserved communities, especially women, with information and services around recommended practices. However, where societies are socially stratified, differences in ethnic identities between agents and beneficiaries may impact the effectiveness of information and service delivery and the uptake of recommended behaviors. We explore the salience of shared ethnic identity between agents and beneficiaries in promoting collective action using a field experiment with women’s self-help groups (SHGs) in India. We cross-randomize an information treatment and a group-agent shared ethnicity treatment at the SHG level. We measure impacts on individual group member information retention and willingness to contribute to a group-owned kitchen garden that could improve access to a diverse and nutritious diet. We find information retention is better when the group is matched with an agent lower in the ethnic hierarchy, but that agents higher in the hierarchy elicit greater individual contributions to the group-owned kitchen garden. We suggest some hypotheses for these seemingly contradictory results. Other characteristics like education, group cohesion and perceived agent ability also matter in changing knowledge and contribution. Our findings have important implications for effective program design and implementation, suggesting that implementers need to consider factors beyond the information content, target group and pedagogical mode of delivery for their strategies to be transformative.

## Highlights

• Many development interventions rely on last-mile agents for information delivery.
• The ethnicities of beneficiaries or agents could affect intervention effectiveness.
• We test this using a field experiment with women’s groups in India.
• Agents deliver information and elicit willingness to contribute to club good.
• Ethnicity matters for information retention and individual contributions.

## 1 Introduction

The importance of integrating information into development programs to affect behavior change is well acknowledged, especially in agriculture, health, and nutrition interventions (Lamstein et al., 2014, Kennedy et al., 2018, Bhutta et al., 2013, Caulfield et al., 1999, Fabrizio et al., 2014, Hoddinott et al., 2017, Ahmed et al., 2016). These programs routinely include the delivery of information (extension) or social and behavior change communication (SBCC) through trained, dedicated agents, with or without in-kind or cash transfers. However, interventions that provide information often emphasize getting the content, target platform and pedagogical mode of delivery right, and in the process, overlook other factors that could limit the effectiveness of the intervention.

In many agriculture and health-centric development programs, individuals from within the community are deployed as agents for information delivery, a decentralized method that leverages existing social networks and agents’ familiarity with local communities and contexts. This is especially true of India, where government and NGO-led agriculture, health and nutrition programs rely on ‘frontline workers’ or agents for last-mile information and service delivery to households. These agents are tasked with ensuring agricultural extension and health and nutrition information and services reach underserved areas and groups, including women (Glendenning et al., 2010, Krishnan, 2022). The role these agents play in bridging last-mile service delivery gaps is particularly important given the slow improvement in both agricultural productivity and health and nutrition indicators in India over the last decade or more (Gillespie et al., 2012, International Institute for Population Sciences (IIPS) and ICF, 2022).

The motivation for this paper is recognition of the fact that the effectiveness of information provision in changing knowledge and behaviors is predicated not only on the quality and accuracy of the information or on the choice of delivery platform, but also on the nature and quality of the interaction between agents and beneficiaries (Golub and Jackson, 2012). Implementing information and behavior change-based campaigns at scale thus requires an investigation into how impacts may be constrained by factors beyond the content of the information.

We investigate the role of one such factor, shared ethnic identity, in determining the impact of information provision on knowledge retention and cooperative action using a lab-in-the-field experiment in the eastern Indian state of West Bengal. We leverage existing women’s self-help groups (SHGs)- savings and credit groups of 10–20 women- to test the impact of information provision and shared ethnic identities between group members and agents on information retention and the willingness of group members to contribute labor towards a group-owned good. The good in question is a kitchen garden, maintained and managed by the SHG and designed to produce a quantity of nutritious fruits and vegetables sufficient to meet the needs of group members and their families for one year.

SHGs are generally ethnically homogenous (see Section 2), so without much loss of generality we retain only those SHGs supported by the partner NGO where all women are of the same ethnic group. Close to 240 SHGs across 88 villages are included in our experiment. We vary the ethnicity of the agent assigned to the group, resulting in matched group-agent pairs where the group is either the same ethnicity as the agent, or higher or lower in the ethnic hierarchy. The ethnic identity of the agent is made salient through their introduction, where they clearly state their full name, a marker of their ethnic affiliation. Agents perform two tasks. First, they provide scripted information to a subset of randomly selected groups in which they motivate the need to improve diet quality and describe the potential role of a group-owned kitchen garden in meeting household fruit and vegetable needs. Second, they conduct a simple exercise to elicit individual willingness to contribute (WTC) labor hours towards the kitchen garden from members of all the groups they meet with, regardless of information provision. In the WTC exercise, members place beans equal to the number of hours per week they are willing to work on the group-owned kitchen garden into a bag; this is done privately and without discussion. Groups that do not receive the information treatment receive a short introduction and then participate in the WTC exercise.

We use this experimental set-up to ask two distinct research questions. First, how does the provision of information affect an individual’s knowledge and their WTC to a group-owned club good? Second, how does the ethnic identity of the agent moderate this relationship? Is information retained better or is it more effective in driving WTC when the agent delivering the message is of the same ethnicity as the group, or when agent is placed higher (or lower) in the social hierarchy? We measure individual knowledge with the percentage of correct responses to a short module based on key points in the scripted information, and WTC by a count of the number of beans the respondent places in the bag.

We find that an individual’s willingness to contribute labor hours to the kitchen garden depends on factors beyond a shared group identity. Agents from the low-ethnic-group elicit lower willingness to contribute, especially when matched with high-ethnic group members. We show that this is not because higher-ethnic-group agents provide more information or are better able to motivate the need for the good. In fact, when combined with an information treatment, individual retention of information is better when the group is paired with a low-ethnic-group agent. We hypothesize that the increased WTC when paired with a higher-ethnic-group agent is instead a response to the perceived ability of the agent to facilitate the provision of the club good.

Several aspects of our experimental setup make our findings relevant to policy discourse in India. First, SHGs are ubiquitous, both with the government-supported National Rural Livelihoods Mission SHGs covering more than 80 million households across the country (NRLM, 2021), and increasingly being used as platforms to deliver agricultural extension and behavior change communication on sanitation, health and nutrition at scale, and to increase awareness and community monitoring of government schemes (Desai et al., 2020, Dìaz-Martin et al., 2020). The centering of these groups in our experiment makes our results of broad relevance.

Second, the choice of the group-owned good as a kitchen garden that enables local provisioning of higher quality diets serves a clear community need. Cross-sectional data from related work in the same study areas in 2015-16 shows that only 7% of women in this area meet the minimum dietary diversity requirements of 5 out of 10 food groups, and fewer than 20% report eating any fruit or vitamin-A rich vegetables. These dietary patterns are mirrored in the case of their children. Good quality diets can improve both under- and over-nutrition as well as micronutrient deficiencies (Arimond et al., 2010, Arimond and Ruel, 2004, Grosso, 2019, Hawkes et al., 2019), making them a key policy lever in the Indian context where overweight and obesity have been rising even as undernutrition remains stubbornly high (IIPS & ICF, 2022). The affordability of healthy nutritious diets has been identified as a key constraint to better nutrition outcomes (Gupta et al., 2021, Raghunathan et al., 2021). Leveraging group contributions to the production of nutritious foods allows for cooperation within the group in sharing the burden of labor while simultaneously serving the needs of multiple households.

Third, our investigation of the role ethnic identity plays in such information-based interventions is also important. In India, ethnic identity – that is, one’s caste or tribe affiliation - shapes one’s economic and social life: where you live, who you marry, who you interact with and how, what services you can access, the education you receive, and the jobs you can aspire to. The role of ethnic identity in shaping behavior, including the extent and effectiveness of your interactions with members of other ethnic groups is, therefore, both an economically important and a socially salient issue.

Our group-agent ethnicity pairings serve to ‘prime’ ethnic identity for members of the group, a treatment that we hypothesize could affect group members’ perception of social exclusion and their feelings of trust towards the agent. This in turn could influence information retention and individual willingness to contribute towards the group-owned good. Evidence on the impact of priming identity on economic outcomes is relatively recent but growing rapidly. Of relevance to our study is the literature from different country contexts showing that survey responses can change based on ethnic, gender or racial identity disparities between interviewers and interviewees (Adida et al., 2016 in 14 African countries; Blaydes and Gillum, 2013 in Egypt; Davis et al., 2010 in the United States; Cilliers et al., 2015 in Sierra Leone). Afridi et al. (2015) show that making one’s *hukou* - the Chinese household registration system - identity salient reduces the performance of rural migrant students relative to urban students. Karachiwalla (2019) uses longitudinal data from Pakistan to show that having a high-caste teacher improves learning outcomes for low-caste children. In India, making caste identity salient can affect children’s test scores and their performance on cognitive tasks and impact both parental and adolescent child aspirations and beliefs about long-run economic outcomes (Hoff and Pandey, 2006, Hoff and Pandey, 2014, Mukherjee, 2015). Having the same caste-identity as a service provider or elected representative can significantly increase a beneficiary’s chance of receiving certain types of public goods or benefits from certain government programs (Kumar and Somanathan, 2015, Berg et al., 2019, Besley et al., 2004).

We add to this body of literature in several ways. First, we exploit two sources of identity to different ends. The implications of the variation in the agent’s ethnic identity to that of the group are discussed above. In addition, working with existing SHGs that have greater levels of cooperation and trust stemming from established group identities and longer-term associations with members of the group, allow us to abstract from issues of intra-group trust and cooperation that could arise with artificial group assignment (see Solow and Kirkwood, 2002, Besley et al., 2004, Goette et al., 2006, Chen and Li, 2009, Kumar and Somanathan, 2015, among others, for discussion of group identities and cooperation).

Second, we investigate the interplay of group identity and a possible reinforcement of (real or perceived) social exclusion through the priming of ethnic identity. In the context of Hispanic enclaves in the US, Candelo et al. (2017) show that the perception of social exclusion can reduce contributions towards public goods. In India, there is a long history of caste discrimination and caste-based segregation resulting in restricted access to public or group-owned goods and other deprivations (see, for example, Gupta, 2000, Shah et al., 2006, Thorat, 2017). Even today, reports in the popular press of caste-based restrictions on access to public goods and services, like sources of water or midday meals in schools, are common. In our setting where an agent delivers information about a group-owned good and then solicits contributions, perceived power disparities, and in-group and out-group trust, and fear of punitive action could significantly affect individual behavior (Wilkes and Wu, 2018, Hoff et al., 2011).

## 2 Context

Women’s groups have emerged as important platforms for improving the economic, political and social empowerment of poor women in a number of countries (Meinzen-Dick et al., 2014, Brody et al., 2017). In India, the most common type of women’s groups, SHGs, have proliferated over the last three decades and are now a central component of many rural development programs. A typical SHG consists of 10–20 women who live in close proximity and meet regularly to deposit money into a group account from which individual loans are provided on a rotating basis or to those in need (Nair, 2005). These groups are generally ethnically homogenous, either organically through self-selection and spatial considerations (villages in India tend to be divided into ethnically homogeneous hamlets, so women living close to one another are typically of the same ethnicity) or through imposition by supporting organizations in the belief that homogeneity improves group cohesion and hence, group performance (Deshmukh-Ranadive, 2004, Sharma, 2001, Baland et al., 2011). This implies that development programs that use SHGs as platforms to deliver interventions may be susceptible to limitations imposed by society’s identity-related conventions, such as the exclusion of lower castes or ethnic groups from certain occupations, public spaces, or public goods.

SHGs are increasingly engaging in a wider portfolio of activities designed to enhance individual member, group or community objectives through collective action (Chen et al., 2006, Desai and Joshi, 2014). For example, many SHGs receive training and inputs to pursue income-generating livelihoods activities, especially in agriculture. With support from the Ministry of Agriculture, SHG members are forming larger collectives like producer companies or farmer producer organizations to improve women farmers’ access to input, output and service-related markets. SHGs are also being used as platforms to deliver health, sanitation and nutrition-related information (Desai et al., 2020).

We collaborated with Professional Assistance for Development Action (PRADAN), an NGO that has worked to form and strengthen SHGs since the 1980s. In addition to their core focus of savings and credit and support to agriculture and livelihoods for income generation, in 2015 PRADAN began providing health and nutrition SBCC to SHG women in select geographic areas through dedicated community agents. An agent is typically a woman from the same village as the SHGs she works with. She receives training in health and nutrition, and then disseminates this information in an SHG meeting through a combination of oral, visual, and participatory methods. The results of this experiment are immediately relevant to the scale-up of PRADAN’s nutrition interventions, while also being informative for other similar interventions led either by government bodies, or NGOs.

We conducted this experiment with PRADAN in Baghmundi block of Purulia district in West Bengal. PRADAN did not pilot its nutrition interventions in Baghmundi, so there is no concern of information spillovers. In addition, the average SHG in this block was 6–7 years old at the time of our intervention, a period long enough for members to have developed intra-group norms of cooperation and trust.

## 3 Study design

**Fig. 1:**
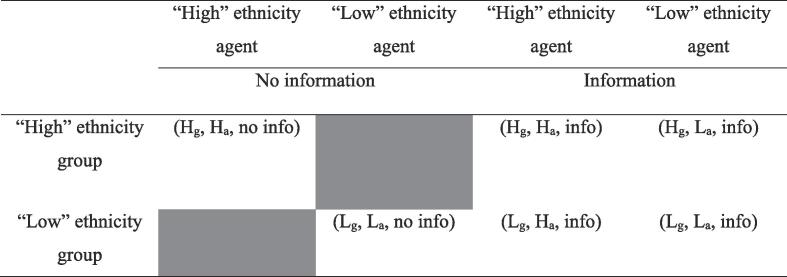
Graphical depiction of the six treatment groups.

We cross-randomize two treatments, the provision of information (“information” treatment), and the ethnic identity of the agent relative to the group (“ethnicity” treatment), depicted in the cells of Fig. 1 by info/no info and the letters H/L for “High”/“Low” ethnic categories respectively. The subscript *g* on an ethnicity variable refers to the group, while the subscript *a* refers to the agent. Thus, treatment (H_g_, H_a_, info) indicates a High ethnicity group paired with a High ethnicity agent who conducts the WTC game and provides information. Similarly, (L_g_, H_a_, no info) indicates a Low ethnicity group paired with a High ethnicity agent who only conducts the WTC game but does not provide any information. The randomization followed a stratified design, with stratification based on ethnicity of the group.

In consultation with PRADAN, we developed a script for the “information” treatment (see Appendix A1.1). The script introduced the agent by name, crucial for identification of ethnic group, motivated the problem of undernutrition in the area, discussed the importance of diverse diets, including the role of different food groups, and ways to improve diet quality, and finally, introduced the concept of the club good, a kitchen garden, and its collective management. The “no information” treatment had a shorter script that introduced the agent by name, without any reference to nutrition, health and diets or motivation of the importance of kitchen gardens (see Appendix A1.2).

Agents received an intensive three-day training, at the end of which they could recite the scripted information from memory. Scripted sessions were key to ensuring that message retention and delivery did not differ substantially across agents and treatment arms. Each information session took about an hour to conduct and was held in a public building, like a school or health center, and care was taken to ensure only group members were present. Over the course of the experiment, each agent conducted both information and no information sessions with different groups, and met with groups of the same or different ethnicity as herself. The information treatment to be conducted with a specific group was conveyed to the agent prior to the group meeting by PRADAN, and reinforced by the independent survey firm.

Within the information treatment, two sets of groups receive information from an agent of the same ethnicity ((H_g_, H_a_, info) and (L_g_, L_a_, info)), while two sets of groups receive information from an agent of a different ethnicity ((H_g_, L_a_, info) and (L_g_, H_a_, info)) (see Fig. 1). We refer to the two sets of groups that are matched with agents of the same ethnicity and that do not receive information – (H_g_, H_a_, noinfo) and (L_g_, L_a_, noinfo) - as the “controls” since they are exposed to neither the ethnicity nor information treatments.

The groups were stratified based on ethnicity and we chose Other Backward Classes (OBCs) and Scheduled Tribes (STs) as the two ethnic groups for our experiment. In Baghmundi, Scheduled Castes (SCs), the other major ethnic group, fall somewhere between the OBCs and STs on the ethnic hierarchy (Appendix Table A2.1), making them less starkly distinct from either OBCs or STs. OBCs are perceived to be higher in the ethnic hierarchy than STs. For the rest of this paper, therefore, the “High” and “Low” ethnic categories refer to OBCs and STs respectively.

Once the agent delivered her script, she engaged group members in the WTC exercise where she asked them to indicate the number of hours they would be willing to devote towards the group-owned club good, a kitchen garden. All SHG members participated in this exercise regardless of their information and ethnicity treatment assignments. At the start of the meeting each SHG member was given two opaque bags, one empty and one containing beans. At the end of the meeting she was asked to transfer to the empty bag a number of beans equal to the number of hours per week she was willing to work on the kitchen garden. This was done privately and without consultation.

The choice of a kitchen garden as the group-owned club good was motivated by several considerations. Kitchen gardens are consistent with PRADAN’s thematic focus on agriculture and related activities, and group members are used to discussing similar topics in SHG meetings. PRADAN uses voluntary labor contributions for agriculture-related activities and for the construction and maintenance of other community assets as well. The community kitchen garden similarly required minimal input besides members’ labor. Most respondents in our sample were staple crop growing farmers, with paddy and coarse grains grown in the two main agriculture seasons, and vegetables grown in between cropping cycles. This meant that the supply of vegetables from their own farm was not consistently available throughout the year. The group kitchen garden was designed to provide reliable year-round supply of seasonal vegetables, with shared time and effort, and the kitchen gardens served a clear community need. In addition, non-group members could be excluded from the produce, making this a classic club good. Women in our sample were already spending on average, 10 or more hours on work, excluding care responsibilities, emblematic of acute time poverty. This was the motivation behind employing a model that leverages group labor in a staggered manner. Finally, clear informational content could be designed around the usefulness of the garden, allowing us to test members’ retention of information provided.

Appendix Fig. A2.1 presents the kitchen garden model designed by PRADAN based on the average number of members per SHG, an average household size of four members and estimated nutritional needs for one year. The total cost was estimated to be INR 10,960 (∼USD 170) per garden, and the total number of labor hours required to build and maintain a garden was estimated at 928 hours per year. This came to approximately 93 hours per year per member, or about 2 hours per member per week. This number was not revealed to the groups at any point. Given that labor was the main recurring input, we elicited WTC labor hours rather than the more traditionally employed willingness to pay. To ensure incentive compatibility, groups were told that the kitchen garden would be implemented in a random selection of groups that had a collective WTC greater than the number of hours required to construct and maintain the garden, and that the cost of non-labor components would be covered by the researchers. Almost all groups had a collective WTC greater than the minimum number of hours needed per week.

It bears mentioning that we were constrained both by available resources and by the total number of existing groups of each ethnic category, and could not fully cross-randomize both treatments, as depicted by the greyed-out section of Fig. 1. This has implications on our ability to estimate causal impacts, to be discussed in further detail in Section 4.

More details about our scoping visits to Baghmundi and our motivation for several design-related decisions are provided in Appendix A3.

### 3.1 Conceptual framework and hypotheses

For the information treatment, the direction of hypothesized impact on both knowledge and WTC is relatively straightforward. All else equal, we expect that providing information on the causes and consequences of undernutrition and introducing the importance of kitchen gardens would increase respondent women’s knowledge, and that this increased knowledge would increase the value SHG members assign to the kitchen garden and hence the number of hours they are willing to contribute to its management.

The interactive effects of the information and ethnicity treatments on knowledge and WTC are more complicated and hypothesized relationships could go in either direction. Information from an agent higher in the ethnic hierarchy could be perceived as being of higher quality, either because the agent has greater education or other qualifications, or is more confident, or because they are perceived to have better access to information. Agents lower in the ethnic hierarchy might be perceived to have lower quality information for analogous reasons. Alternatively, members of the low ethnic group so rarely occupy a position where they deliver information to or facilitate tasks with members of higher ethnicities^1^ that the “novelty” factor could cause high-ethnicity group members to pay closer attention and retain more information. Finally, the priming of ethnicity in the context of existing inter-ethnic-group power dynamics could lead to low-ethnicity group members mistrusting information provided by an agent who belongs to a higher ethnic group than them. The overall impact of the ethnicity treatment on information retention is ambiguous.

Similar considerations apply in the case of WTC towards the group-owned kitchen garden. On the one hand, groups paired with agents of a higher ethnicity than them might want to please the high-ethnicity agent or fear social sanction if they do not cooperate, causing them to increase their WTC. On the other hand, if the information these agents provide is viewed with suspicion, group members might be reluctant to contribute labor hours, reducing their WTC.

These relationships are further complicated in this experiment where we prime ethnicity and solicit contributions towards a good from which members of certain groups can be excluded. A desire to exclude out-group members could increase WTC when respondents assume an affinity with a similarly placed agent; conversely, perceived social inclusion could serve to reduce contributions, as in Candelo et al. (2017).

Finally, it is plausible that individual WTC varies depending on the group members’ beliefs about whether the agent would facilitate the construction of the group-owned good in question. Agents were not explicitly identified as providing additional inputs or resources, but certain portions of the scripted information (see Appendix A1.1 and A1.2) could be interpreted as requiring the agents’ assistance. For example, the script contains the following text: *“As a group you can collectively lease a plot of land, and on this land, you can grow all kinds of fruits and vegetables.”* and *“Other than a small investment to procure seeds and saplings, there is no monetary payment required to participate in this kitchen garden. Instead of money, you all can invest hours of your own time to maintain and grow the garden.”* If the group believed that the agent would help identify land that could be leased or provide the resources for this land or for the other start-up inputs, this could affect their WTC as well. A high-ethnicity agent might be perceived as being more able to deliver on these complementary inputs than a low-ethnicity agent, due to their improved networks, thereby driving WTC up. But agents of a different ethnicity than that of the group might be viewed as being less likely to work to supply the inputs needed for the good, thereby driving WTC down.

These hypothesized relationships are not exhaustive. Our point is simply to illustrate that there are multiple possible mechanisms often operating in opposition to one another, making the question of the final impact of shared ethnic identities on WTC one of empirical interest.

## 4 Data and empirical framework

### 4.1 Sample

We used PRADAN’s internal monitoring data to obtain the full list of SHGs in Baghmundi along with their membership and ethnic composition.^2^ We restricted ourselves to those groups that were entirely OBC or entirely ST, eliminating all groups of mixed ethnicities. Groups of homogenous ethnic composition made up more than 75% of the complete list of SHGs in Baghmundi, so restricting ourselves to such groups is unlikely to affect the generalizability of our results. We then selected 40 groups per treatment arm, for a total of 240 SHGs spread over 88 villages. Based on the MIS data, these 240 SHGs comprised 2790 individual members. Of the 88 villages in our sample, 37 villages had only treatment groups, 10 only had control groups, the remaining 41 had a mix of treatment and control villages. The number of selected groups within these villages ranged from 1 (32%) to 10 (1.1%), and more than 70% villages had three or fewer groups. Each agent in the study was assigned 9-12% of the total number of groups (that is, 20-30 groups each), with a mix of both OBC and ST groups, regardless of their own ethnicity.

We selected nine agents from the same two ethnic groups to deliver the information treatment and conduct the WTC exercise. Of these, five agents were OBC and four ST. Agents were chosen from the pool of Community Data Collectors, cadres of women with some basic educational qualifications that PRADAN engages for routine internal data collection purposes. Barring 63 respondents, all others were matched with an agent belonging to a different village than their own. Agent selection followed no criteria other than their availability and willingness to participate.

### 4.2 Survey

As soon as the agent-led meeting described in Section 3 ended, the SHG women left the venue with their bags from the WTC exercise, and were interviewed by enumerators hired by an independent data collection firm. One enumerator was assigned to each respondent and the surveys were conducted in parallel, thereby eliminating the possibility of women conferring with others in their group before being interviewed. The survey asked the respondent the number of labor hours she volunteered and checked this against the number of beans in the bag. In case of a discrepancy, the enumerator elicited the ‘final’ response and recorded all three responses.

The survey also collected information on basic demographic and socioeconomic characteristics, women’s time use and opportunity cost of labor, attitudes towards group members and concepts of trust, perception of the agent (including a question about identification of the agent’s ethnicity), and market access, among other things. All respondent women were administered a ten-question nutrition knowledge test based on the content of the scripted information session (Appendix A4). They received a score of one for each correct answer, and the unweighted sum of scores across all ten questions was used as a measure of knowledge retention.

One concern is that individuals within a group might be able to confer, allowing them to jointly determine their willingness to contribute labor hours. Given the way the survey and experiment were executed, we believe this is not a significant concern. The intervention was conducted in an enclosed public space, a school or health center. As soon as the participants left the session, each respondent was met by one enumerator and interviews were conducted in parallel, thus ensuring that the gap between the intervention and the individual surveys was kept to a minimum. A second concern could be contamination across treatment and control groups within a village. While this is possible, it is unlikely. The intervention and interview sessions were conducted either in parallel or in quick succession with the different groups in the village, and given that 70% of the villages had three or fewer groups, each village took two days to cover (at most, and typically only a single day). Finally, since different villages were surveyed on different days, the concern of cross-village contamination is valid. However, we believe this risk is also small, since the survey plan was designed to minimize survey costs, meaning that contiguous villages were visited within a short span of time. The window in which the resident of one village could visit a nearby village and discuss the intervention or the survey interview was in the range of 1–3 days. It also bears noting that large parts of our study area are remote, hilly and forested, with villages spatially spread out, making it more unlikely that residents of one village made an impromptu visit to another nearby village to discuss the intervention.

Finally, members of the research team conducted a survey with the agents to collect information on their demographics, education, height, self-confidence, self-esteem and work history.

#### 4.2.1 Ethical considerations

Ethical approval for this study was provided by the Institutional Review Board of the researchers’ institute. Enumerators sought informed oral consent before the start of each interview. Respondents were informed of the broad goals of the study, that their data would be kept private and confidential and that they had the right to withdraw consent at any point during the interview.

### 4.3 Econometric specification

The basic estimating equation for the outcomes of interest for individual *i* belonging to SHG *g* in village *v* is

(1) $$Y_{\mathrm{igv}}=\alpha +\beta_{1}AGENT_{\mathrm{gv}}+\beta_{2}INFO_{\mathrm{gv}}+\beta_{3}AGENT_{\mathrm{gv}}*INFO_{\mathrm{gv}}+\beta_{4}Z_{\mathrm{igv}}+\beta_{5}W_{\mathrm{gv}}+{∊}_{\mathrm{igv}},$$

where $Y_{\mathrm{igv}}$ refers to the outcome variable: knowledge score (in points) and WTC (in hours). $\mathrm{AGEN}T_{\mathrm{gv}}$ is the type of agent assigned to SHG *g* in village *v*. $\mathrm{INF}O_{\mathrm{gv}}$ is a binary variable indicating whether SHG *g* in village *v* received the information treatment. $Z_{\mathrm{igv}}$ are individual level covariates (see below). $W_{\mathrm{gv}}$ is the number of SHG members present for the experiment, and ${∊}_{\mathrm{igv}}$ is an individual-SHG level error term, clustered at the SHG-level. The small number of SHGs in some village precludes the use of village fixed effects in these regressions. We use ordinary least squares to estimate the coefficients of interest, and variants of equation (1) to identify additional impacts.

We control for two sets of individual characteristics, with the base category for binary variables indicated in parentheses. First, a ‘restricted’ set that are likely to be independent of the treatment, which includes respondent age in years, marital status (not married), ethnicity (ST), religion (Hindu), employment status (unemployed), education categories, household size, number of children under five, wealth quintiles based on asset ownership^3^, total land cultivated, and duration of SHG membership. Second, a ‘full’ set of characteristics that adds to the restricted set, variables whose values may have been impacted by either of the treatments. These include the total number of food groups consumed by the respondent woman in the 24 hours preceding the survey, respondent currently has a kitchen garden (no), respondent grows vegetables on their land (no), respondent agrees that produce from the kitchen garden should be split equally among group members (no), number of completed hours spent on work in a day based on a 24-hour recall time-use survey module, self-reported group cohesiveness and individual perceptions of agent’s ability (see below for details on variable construction).

Missing covariate values were replaced with 0s and flags for missing values were added, so that our analysis was conducted on a balanced sample of women. This approach is unlikely to introduce bias since only four variables had missing values, and the proportion of missing values was relatively low. These variables (with the proportion missing in parentheses) are employment status (0.63), total land cultivated (0.22), currently have a kitchen garden (17.63) and number of completed hours spent on work in a day based on a 24-hour recall time-use survey module (0.04).

Two caveats are worth mentioning in detail here. First, as alluded to above, we were unable to fully cross-randomize our two treatments. In the absence of full cross-randomization, our estimates of impact in the full sample are likely to be biased. However, conditional on having received information, our randomization is complete. This allows us to analyze the impact of shared or disparate ethnic identities between the group and the agent for the subset of groups that received the information treatment. We temper all causal claims in our discussion of the results below.

Second, since agents were selected from among the same villages as the SHGs, there is a concern that close familiarity with the agent could be driving some of our results. There are only 63 respondents in our sample who were matched to an agent who belonged to the same village. Since this is such a small number, it is perhaps unsurprising that our results do not change when these individuals are dropped from our sample. For brevity, we report results using the full sample for all the following tables.

#### 4.3.1 Group cohesion

Individuals were asked four questions regarding the cohesiveness of the group they belonged to:

(1) Do you trust other members of your group to make decisions that are in the best interest of the whole group?
(2) Do you trust other members of your group to make decisions that are in your best interest?
(3) During the last six months, did your SHG collectively or along with other community members demand any entitlements related to health from government frontline workers? and
(4) In the past six months has there been any instance where SHG members came together to negotiate for their rights at a village facility?

Each of these questions was scored 1 if the respondent said yes and 0 otherwise, and scores added together to construct a measure of group cohesiveness. Fig. A2.2 presents the distribution of the resulting measure; close to three-fouths of the sample report a score of 2 on 4.

#### 4.3.2 Perception of agent quality

Respondents were asked six yes–no questions about their perception of the agent:

(1) The agent communicated messages clearly and patiently until all members understood them,
(2) The agent was an engaging speaker and was able to keep the attention of the women,
(3) The agent summarized important actions to be taken at the end of the meeting,
(4) Do you think the agent has the ability to facilitate the construction of the kitchen garden she talked about,
(5) Do you trust the agent to act in the interest of your SHG, and
(6) Compared to yourself, do you think the agent knows more about nutrition and kitchen gardens.

Each of these questions was scored 1 if the respondent said yes and 0 otherwise, and scores added together to construct a measure of agent ability that ranged from 0 to 6. Fig. A2.3 presents the distribution of this measure. More than half the sample said yes to all six questions.

A couple of observations are worth noting before we turn to the results:

First, based on our available budget, kitchen gardens were subsequently implemented in six groups. This implies that the odds of a group being selected for kitchen garden construction were small (6/240 = 2.5%). To elicit truthful revelation of the WTC these odds were not conveyed to group members.

Second, we did not specify a mechanism by which a group member would be held to their committed number of hours. This was done for two reasons. First, groups had existing mechanisms to determine member contribution in other scenarios, and discussions during the pilot visit suggested that groups would prefer to employ their own monitoring and enforcement techniques. Second, the total contributions of almost all groups exceeded the hours needed. Unfortunately, we did not collect information on members’ expectation regarding enforcement, or their beliefs around other members’ contributions. In the absence of unambiguous enforcement mechanisms, we acknowledge that group members might inflate their contributions.

## 5 Results

We analyze the effect of information provision and shared identity on our main outcomes of interest: an individual’s knowledge scores on the nutrition knowledge test and their WTC labor hours to the group-owned kitchen garden. We begin by presenting some descriptive statistics on the various group-agent-information combinations, and then proceed to discuss the regression results by specification.

### 5.1 Descriptive statistics

Of the 2790 SHG members in the set of SHGs selected to be part of the study, only 2238 were available for the experiment and interview, and gave complete responses. Of these, 15 women were dropped from our sample due to missing data on the main dependent variables. Our final sample was 2223 SHG members from 240 SHGs in 88 villages.

Table 1 presents individual, household, and SHG level characteristics for each of the six treatment arms. A priori we expect balance within groups, across treatment arms for the restricted set of variables, and we find that holds for most variables. Differences between high ethnicity and low ethnicity groups are expected and observed. The average respondent woman is between 38 and 39 years old, more than 80% of the women in our sample are married and more than half do not have any formal education. The proportion with no formal education is considerably higher among ST women. Between 23 and 32% of the women in our sample are employed and consumed only 3–4 food groups out of a possible 10 in the preceding 24 hours. On average, low ethnicity group members consume fewer food groups than high ethnicity members. An average household owns 6–8 assets out of a possible 24, has close to five members and owns 1 acre of cultivable land. About 33–39% of women in our sample have a kitchen garden in their household while about 39–51% grow vegetables on their farm. Women have been associated with their group for 79–96 months, or 6–8 years. Most members perceived their group to be moderately cohesive, but the perceived ability of the agent was high, more than 5 out of a possible maximum score of 6.

**Table 1:** Descriptive statistics for study sample.

	H_g_,H_a_,noinfo	H_g_,H_a_,info	H_g_,L_a_,info	L_g_,L_a_,noinfo	L_g_,L_a_,info	L_g_,H_a_,info	Total
Variable	Mean/SE	Mean/SE	Mean/SE	Mean/SE	Mean/SE	Mean/SE	Mean/SE
	(1)	(2)	(3)	(4)	(5)	(6)	(7)
Age of resp. woman	39.40	38.78	39.66	38.90	39.54	38.79	39.18
	[0.58]	[0.59]	[0.56]	[0.69]	[0.68]	[0.64]	[0.25]
Respondent is currently married ^c, d, e, g, h, i, j, k, l^	0.89	0.89	0.90	0.84	0.81	0.83	0.86
	[0.02]	[0.02]	[0.02]	[0.02]	[0.02]	[0.02]	[0.01]
No formal education ^c, d, e, g, h, i, j, k, l^	0.49	0.53	0.53	0.71	0.71	0.72	0.61
	[0.03]	[0.03]	[0.03]	[0.02]	[0.02]	[0.02]	[0.01]
Primary (up to and including class 5) ^c, d, g, h, j, n^	0.23	0.24	0.21	0.15	0.17	0.19	0.20
	[0.02]	[0.02]	[0.02]	[0.02]	[0.02]	[0.02]	[0.01]
Middle school (classes 6–8) ^a, c, d, e, f, i, j, k, l, o^	0.12	0.08	0.13	0.05	0.06	0.04	0.08
	[0.02]	[0.01]	[0.02]	[0.01]	[0.01]	[0.01]	[0.01]
Secondary or above ^c, d, e, g, h, i, k, l, m, n^	0.16	0.16	0.14	0.10	0.06	0.06	0.11
	[0.02]	[0.02]	[0.02]	[0.02]	[0.01]	[0.01]	[0.01]
Respondent woman is employed ^c, d, e, g, h, i^	0.22	0.24	0.27	0.31	0.31	0.30	0.28
	[0.02]	[0.02]	[0.02]	[0.02]	[0.02]	[0.02]	[0.01]
Total number of food groups consumed by women ^a, c, d, f, g, h, i, j, k, l, m, n, o^	4.02	4.22	4.05	3.19	3.49	3.88	3.82
	[0.07]	[0.07]	[0.07]	[0.06]	[0.07]	[0.06]	[0.03]
Assets (sum) ^b, c, d, e, f, g, h, i, j, k, l^	8.66	8.76	8.13	6.37	6.38	6.30	7.46
	[0.14]	[0.15]	[0.16]	[0.13]	[0.12]	[0.12]	[0.06]
Household size ^o^	4.72	4.78	4.80	4.73	4.85	4.62	4.75
	[0.09]	[0.10]	[0.09]	[0.10]	[0.10]	[0.09]	[0.04]
# of children under 5 years ^d, h, j, k, l, m, o^	0.38	0.34	0.31	0.41	0.49	0.40	0.39
	[0.03]	[0.03]	[0.03]	[0.03]	[0.04]	[0.03]	[0.01]
Total land cultivated (in acres) ^d, e, h, i, k, l, m^	1.18	1.21	1.11	1.05	0.87	0.93	1.06
	[0.07]	[0.08]	[0.06]	[0.08]	[0.05]	[0.05]	[0.03]
Time spent working (in completed hours^) a, b, c, d, e, g, i, j, l, m, o^	10.16	9.80	9.87	10.28	9.84	10.50	10.08
	[0.13]	[0.13]	[0.13]	[0.13]	[0.13]	[0.13]	[0.05]
Currently have home garden ^a, c, d, e, j^	0.37	0.31	0.33	0.27	0.28	0.29	0.31
	[0.02]	[0.02]	[0.02]	[0.02]	[0.02]	[0.02]	[0.01]
Grows vegetables on farm ^a, e, f, g, l, m^	0.50	0.40	0.48	0.49	0.45	0.39	0.45
	[0.03]	[0.03]	[0.03]	[0.03]	[0.03]	[0.03]	[0.01]
Duration of SHG membership (years) ^c, d, g, j, k, n^	6.31	6.63	6.53	7.82	7.19	6.84	6.87
	[0.23]	[0.27]	[0.27]	[0.27]	[0.29]	[0.24]	[0.11]
# group members present for experiment ^a, c, d, g, h, j, k, m, n, o^	10.20	9.81	9.96	9.42	9.10	9.99	9.76
	[0.13]	[0.15]	[0.14]	[0.13]	[0.07]	[0.13]	[0.05]
Agree that produce should be split equally ^b, c, d, e, f, g, h, l, n, o^	0.81	0.77	0.65	0.65	0.67	0.73	0.72
	[0.02]	[0.02]	[0.02]	[0.03]	[0.02]	[0.02]	[0.01]
Perceived group cohesion (0–4) ^d^	1.86	1.85	1.84	1.85	1.78	1.85	1.84
	[0.03]	[0.03]	[0.03]	[0.04]	[0.04]	[0.03]	[0.01]
Perceived ability of the agent (0–6) ^a, g, h, i, j, k^	5.23	5.40	5.30	5.15	5.12	5.23	5.24
	[0.06]	[0.05]	[0.06]	[0.06]	[0.07]	[0.06]	[0.02]
N	389	370	379	350	356	379	2223

Notes: Statistically significant differences at 5% across columns are expressed using superscripts on the variable names. Superscripts correspond to column-wise comparisons as follows: a: (1)-(2), b: (1)-(3), c: (1)-(4), d: (1)-(5), e: (1)-(6), f: (2)-(3), g: (2)-(4), h: (2)-(5), i: (2)-(6), j: (3)-(4), k: (3)-(5), l: (3)-(6), m: (4)-(5), n: (4)-(6), o: (5)-(6).

### 5.2 Effect of information and shared ethnicity treatments

We start by looking at how information provision is associated with knowledge retention. Table 2 reports the association of the information treatment and the nutrition knowledge test score, first in the full sample (columns 1–3) and then in the sub-set of groups matched to agents of the same ethnicity (columns 4–6). The first column in the table has no additional controls, and each subsequent column adds a set of covariates.

**Table 2:** Association of the information and ethnicity treatments with the nutrition knowledge score.

	Full sample			Groups matched to same-ethnicity agents		
	(1)	(2)	(3)	(4)	(5)	(6)
Information treatment	−0.05	0.21	0.20	0.39	0.40	0.40
	(0.12)	(0.10)**	(0.10)**	(0.09)***	(0.08)***	(0.08)***
Low ethnicity agent	−0.15	0.33	0.35			
	(0.14)	(0.15)**	(0.15)**			
Information treatment*Low ethnicity agent	0.75	0.28	0.30			
	(0.17)***	(0.16)*	(0.16)*			
*R*^2^	0.03	0.12	0.16	0.01	0.09	0.13
*N*	2,223	2,223	2,223	1,465	1,465	1,465
Mean of Control	7.22	7.22	7.22	7.15	7.15	7.15
Controls		Restricted	Full		Restricted	Full

For columns 1–3 the relevant control group is those SHGs that did not receive information and were matched to a high-ethnicity agent. For columns 4–6 it is those SHGs that were matched to same ethnicity agents and did not receive information. Restricted controls include respondent age, marital status (1/0), ethnicity (=ST), religion (=Hindu), employment status (1/0), education, HH size, number of children under the age of 5 years, wealth quintiles, total land cultivated, number of years they have been an SHG member and the number of SHG members present at the experiment. Full controls additionally include #food groups consumed, have kitchen garden (1/0), grow vegetables on their land (1/0), agree that produce should be split equally (1/0), dummies for group cohesion and for agent ability. Standard errors (in parentheses) clustered at the SHG level. * *p* < 0.1 ** *p* < 0.05; *** *p* < 0.01.

Receiving information has a positive and significant association with the respondent’s nutrition knowledge score, both in the full-sample and in the sub-groups matched with same ethnicity agents. For the sub-group in columns 4–6, the association between information provision and the nutrition knowledge score is 0.40, about 5.5 percent over the mean knowledge score in the control group, those SHGs matched to same ethnicity agents that did not receive information. It would appear from these results that respondent women are able to understand and retain the information provided, at least in the short term.

From rows 2 and 3 of Table 2 we also see that being matched with a low ethnicity agent is associated with a significantly higher knowledge score. Note that this captures the correlation between being matched with a low (ST) agent regardless of the group’s own ethnic composition – i.e., this is not predicated on the agent being *lower* in the ethnic hierarchy.

Information appears to be positively correlated with an invididual’s knowledge score, but does improved information and knowledge translate into increased WTC? Table 3 presents the relationship between the information and ethnicity treatments on labor hours contributed. In the full sample (columns 1–3), we find no significant impact of information provision on an individual’s WTC. We do find, however, that information provided by a low ethnicity agent is associated with a significantly lower individual WTC, varying in magnitude from 3.6 to 4.5 hours per week depending on the model specification (p < 0.01). For the full specification (column 3), this is 30% lower relative to the mean in the relevant control group, a significant and qualitatively large coefficient. As in Table 2, Columns 4–6 restricts the sample to those groups that are matched to agents of the same ethnicity. In this sub-sample, we find no relationship between information provision and willingness to contribute.

**Table 3:** Association of the information and ethnicity treatments with willingness to contribute labor hours.

	Full sample			Groups matched to same-ethnicity agents		
	(1)	(2)	(3)	(4)	(5)	(6)
Information treatment	−1.37	−0.94	−0.88	−0.45	−0.29	−0.25
	(1.23)	(1.25)	(1.18)	(0.85)	(0.77)	(0.74)
Low ethnicity agent	−4.50	−3.88	−3.60			
	(1.16)***	(1.31)***	(1.25)***			
Information treatment*Low ethnicity agent	1.70	1.08	0.79			
	(1.37)	(1.49)	(1.43)			
*R*^2^	0.06	0.08	0.11	0.00	0.10	0.14
*N*	2,223	2,223	2,223	1,465	1,465	1,465
Mean of Control	11.76	11.76	11.76	9.62	9.62	9.62
Controls		Restricted	Full		Restricted	Full

For columns 1–3 the relevant control group is those SHGs that did not receive information and were matched to a high-ethnicity agent. For columns 4–6 it is those SHGs that were matched to same ethnicity agents and did not receive information. Restricted controls include respondent age, marital status (1/0), ethnicity (=ST), religion (=Hindu), employment status (1/0), education, HH size, number of children under the age of 5 years, wealth quintiles, total land cultivated, number of years they have been an SHG member and the number of SHG members present at the experiment. Full controls additionally include #food groups consumed, have kitchen garden (1/0), grow vegetables on their land (1/0), agree that produce should be split equally (1/0), dummies for group cohesion and for agent ability. Standard errors (in parentheses) clustered at the SHG level. * *p* < 0.1 ** *p* < 0.05; *** *p* < 0.01.

Given that information is associated with improved knowledge on average, the lack of any association between information and willingness to contribute among homogenous groups matched with the same ethnicity agent as themselves suggests that the message alone is not sufficient and other factors might be important in improving willingness to contribute. However, we also see that being matched with a low ethnicity agent is associated with higher knowledge retention but also a lower willingness to contribute. One possible hypothesis that could help reconcile the results in Table 2, Table 3 is that the women are not responding only to the salience of the club good, but also to their confidence in the ability of the agent to deliver the good in question. As mentioned above, even though the scripted information does not explicitly say that the agent will help deliver the good in question, certain phrases within it could imply that the agent’s help would be needed to facilitate construction. It is possible that having a low-ethnicity agent address the SHG and deliver information is novel enough for women to pay keen attention to what they are saying. However, when it comes time to contribute their labor, the low-ethnicity agent is perceived as less able to follow through on delivery of the public good as a result of fewer resources or limited social capital, thereby prompting women to pledge less. Alternatively, women may volunteer higher labor hours when matched with a high-ethnicity agent because of greater belief in the agent’s ability to facilitate actual construction of the good, or fear of social sanction if they do not cooperate.

Next, we look at the association between being matched with an agent from the same ethnicity as the group and the individual’s knowledge score (Table 4), both for the full sample (Columns 1–3) and the sub-sample of groups that received information (Columns 4–6). In neither case do we find a significant association between being matched to an agent of the same ethnicity and the nutrition knowledge score.

**Table 4:** Association of shared agent-group ethnicity and nutrition knowledge score.

	Full sample			Groups that received information		
	(1)	(2)	(3)	(4)	(5)	(6)
Group matched with agent from same etnicity	−0.05	−0.09	−0.11	0.14	0.13	0.12
	(0.10)	(0.09)	(0.09)	(0.11)	(0.09)	(0.10)
*R*^2^	0.00	0.09	0.12	0.00	0.10	0.14
*N*	2,223	2,223	2,223	1,484	1,484	1,484
Mean of Control	7.39	7.39	7.39	7.39	7.39	7.39
Controls		Restricted	Full		Restricted	Full

For columns 1–3 the relevant control group is those SHGs that were not matched to a same-ethnicity agent. For columns 4–6 it is those SHGs that received information but that were not matched to a same-ethnicity agent. Restricted controls include respondent age, marital status (1/0), ethnicity (=ST), religion (=Hindu), employment status (1/0), education, HH size, number of children under the age of 5 years, wealth quintiles, total land cultivated, number of years they have been an SHG member and the number of SHG members present at the experiment. Full controls additionally include #food groups consumed, have kitchen garden (1/0), grow vegetables on their land (1/0), agree that produce should be split equally (1/0), dummies for group cohesion and for agent ability. Standard errors (in parentheses) clustered at the SHG level. * *p* < 0.1 ** *p* < 0.05; *** *p* < 0.01.

The relationship between shared ethnicity and willingness to contribute labor hours (Table 5) shows a similar lack of association, both in the full sample (Columns 1–3) and the sub-sample that received information (Columns 4–6). Being matched to an agent of the same ethnicity as you does not appear to elicit greater cooperation regardless of whether they provide information. We saw in Table 3 that there was a strong negative association between being matched to a low-ethnicity agent and one’s willingness to contribute. Taken together with these findings, this suggests that the result in Table 3 is driven by high-ethnicity groups matched to low-ethnicity agents.

**Table 5:** Association of shared agent-group ethnicity and willingness to contribute labor hours.

	Full sample			Groups that received information		
	(1)	(2)	(3)	(4)	(5)	(6)
Group matched with agent from same ethnicity	0.57	0.48	0.79	0.34	0.29	0.64
	(0.70)	(0.70)	(0.66)	(0.78)	(0.75)	(0.70)
*R*^2^	0.00	0.04	0.08	0.00	0.03	0.07
*N*	2,223	2,223	2,223	1,484	1,484	1,484
Mean of Control	7.39	7.39	7.39	7.39	7.39	7.39
Controls		Restricted	Full		Restricted	Full

For columns 1–3 the relevant control group is those SHGs that were not matched to a same-ethnicity agent. For columns 4–6 it is those SHGs that received information but that were not matched to a same-ethnicity agent. Restricted controls include respondent age, marital status (1/0), ethnicity (=ST), religion (=Hindu), employment status (1/0), education, HH size, number of children under the age of 5 years, wealth quintiles, total land cultivated, number of years they have been an SHG member and the number of SHG members present at the experiment. Full controls additionally include #food groups consumed, have kitchen garden (1/0), grow vegetables on their land (1/0), agree that produce should be split equally (1/0), dummies for group cohesion and for agent ability. Standard errors (in parentheses) clustered at the SHG level. * *p* < 0.1 ** *p* < 0.05; *** *p* < 0.01.

Are results different when high (or low) groups are matched with low (or high) agents and vice-versa? Table 6 restricts the sample to those who received information and presents the impact of the different ethnicity treatment on the nutrition knowledge score. All results are relative to the omitted group of (H_g_,H_a_, info), i.e., those high-ethnicity SHGs matched with high-ethnicity agents that received the information treatment.

**Table 6:** Association of the ethnicity treatment and nutrition knowledge score among SHGs that received information.

	Nutrition knowledge score		
	(1)	(2)	(3)
Group type (H_g_, L_a_, info)	0.45	0.48	0.54
	(0.11)***	(0.10)***	(0.11)***
Group type (L_g_, L_a_, info)	0.07	0.22	0.43
	(0.11)	(0.33)	(0.34)
Group type (L_g_, H_a_, info)	−0.66	−0.52	−0.37
	(0.13)***	(0.34)	(0.33)
*R*^2^	0.06	0.14	0.18
*N*	1,484	1,484	1,484
Mean of Control	7.5	7.5	7.5
Controls		Restricted	Full

Relevant control group is the set of (Hg,Ha, info) groups. Restricted controls include respondent age, marital status (1/0), ethnicity (=ST), religion (=Hindu), employment status (1/0), education, HH size, number of children under the age of 5 years, wealth quintiles, total land cultivated, number of years they have been an SHG member and the number of SHG members present at the experiment. Full controls additionally include #food groups consumed, have kitchen garden (1/0), grow vegetables on their land (1/0), agree that produce should be split equally (1/0), dummies for group cohesion and for agent ability. Standard errors (in parentheses) clustered at the SHG level. * *p* < 0.1 ** *p* < 0.05; *** *p* < 0.01.

Respondents from high-ethnicity groups matched to a low-ethnicity agent (group type (H_g_, L_a_, info)) display greater retention of information, with a significant coefficient estimate of 0.54 in the fully specified model (Column 3), a 7% increase over the control group mean. Interestingly, we see no positive association with knowledge score for the (L_g_,L_a_, info) group, relative to the base (H_g_,H_a_, info) group. Similarly, being in the (L_g_,H_a_, info) group was statistically indistinguishable from the base, with respect to nutrition knowledge. For the (H_g_,L_a_, info) group, as hypothesized above, what might be at play is the novelty of having a low-ethnicity agent providing information, something that is still rare in practice.

Is this increased knowledge also reflected in WTC? As suspected from the results in Table 2, Table 3, Table 4 above, we find exactly the opposite to be true when looking at WTC labor hours. Table 7 presents results for WTC relative to the base category of (H_g_,H_a_, info) groups. Once again, the (L_g_,H_a_, info) group is indistinguishable from (H_g_,H_a_, info). However, contrary to what one might expect given the strong positive relationship on knowledge score noted in Table 6, the impact on willingness to contribute for (H_g_,L_a_, info) group is large, negative and statistically significant relative to the base. Despite retaining more information and scoring higher on the knowledge score test, high group members are less willing to volunteer labor hours when paired with an agent of low ethnicity. In the next sub-section, we try to unpack some mechanisms driving these results.

**Table 7:** Association of the ethnicity treatment and willingness to contribute labor hours among SHGs that received information.

	Willingness to contribute labor hours		
	(1)	(2)	(3)
Group type (H_g_, L_a_, info)	−3.13	−3.03	−3.40
	(1.00)***	(1.00)***	(0.97)***
Group type (L_g_, L_a_, info)	−3.39	−2.30	−2.49
	(1.02)***	(1.99)	(1.77)
Group type (L_g_, H_a_, info)	−0.89	0.20	−0.30
	(1.33)	(2.32)	(2.07)
*R*^2^	0.04	0.06	0.11
*N*	1,484	1,484	1,484
Mean of Control	10.83	10.83	10.83
Controls		Restricted	Full

Relevant control group is the set of (Hg,Ha, info) groups. Restricted controls include respondent age, marital status (1/0), ethnicity (=ST), religion (=Hindu), employment status (1/0), education, HH size, number of children under the age of 5 years, wealth quintiles, total land cultivated, number of years they have been an SHG member and the number of SHG members present at the experiment. Full controls additionally include #food groups consumed, have kitchen garden (1/0), grow vegetables on their land (1/0), agree that produce should be split equally (1/0), dummies for group cohesion and for agent ability. Standard errors (in parentheses) clustered at the SHG level. * *p* < 0.1 ** *p* < 0.05; *** *p* < 0.01.

### 5.3 Robustness and possible mechanisms

The results presented above could be driven by observable or unobservable agent characteristics, such as their articulateness or ability to project confidence. In addition, our hypotheses about impact are predicated on the assumption that respondents are indeed able to correctly identify the agent’s place on the ethnic hierarchy. We discuss some robustness checks that address both concerns. We also test two possible mechanisms for our results via greater perceived group cohesiveness and agent ability, and its relationship with the treatment.

#### 5.3.1 Agent characteristics

Appendix Table A2.2 presents summaries of agent characteristics across both groups of agents. ST agents are on average younger, more educated and shorter than OBC agents, own fewer assets and score less on self-reported self-esteem. Note that this runs contrary to the comparison of education status among group members, where ST members had significantly lower education levels than OBCs. It is possible that ST agents need to demonstrate higher qualifications to overcome implicit ethnicity related biases.

To account for systematic differences across different agents, we re-run our main specifications by including agent fixed effects (Table 8, Table 9). Information on the agent they were matched to was inadvertently missing for 117 respondents in our sample due to a coding error in the data entry program; in absence of this information, we are left with no option but to drop these observations from the results in these tables. We note, however, that there are no obvious patterns in either group or individual characteristics among this set of 117 individuals that would serve to bias our results in a particular direction.

**Table 8:** Association of the information and ethnicity treatments with the nutrition knowledge score, agent fixed effects included.

	Full sample			Groups matched to same-ethnicity agents		
	(1)	(2)	(3)	(4)	(5)	(6)
Information treatment	0.05	0.26	0.25	0.41	0.44	0.43
	(0.10)	(0.09)***	(0.10)**	(0.09)***	(0.08)***	(0.08)***
Low ethnicity agent	−0.31	−0.02	−0.01			
	(0.21)	(0.20)	(0.21)			
Information treatment*Low ethnicity agent	0.61	0.24	0.26			
	(0.16)***	(0.16)	(0.16)*			
*R*^2^	0.06	0.14	0.18	0.03	0.11	0.14
*N*	2,106	2,106	2,106	1,395	1,395	1,395
Mean of Control	7.22	7.22	7.22	7.15	7.15	7.15
Agent fixed effects	X	X	X	X	X	X
Controls		Restricted	Full		Restricted	Full

For columns 1–3 the relevant control group is all SHGs that did not receive information and were matched to a high-ethnicity agent. For columns 4–6 the control group is those SHGs that were matched to same ethnicity agents and did not receive information. All regressions include agent fixed effects. We exclude the 117 respondents for whom data on agent name is not available. Restricted controls include respondent age, marital status (1/0), ethnicity (=ST), religion (=Hindu), employment status (1/0), education, HH size, number of children under the age of 5 years, wealth quintiles, total land cultivated, number of years they have been an SHG member and the number of SHG members present at the experiment. Full controls additionally include #food groups consumed, have kitchen garden (1/0), grow vegetables on their land (1/0), agree that produce should be split equally (1/0), dummies for group cohesion and for agent ability. Standard errors (in parentheses) clustered at the SHG level. * *p* < 0.1 ** *p* < 0.05; *** *p* < 0.01.

**Table 9:** Association of the information and ethnicity treatments with willingness to contribute labor hours, agent fixed effects included.

	Full sample			Groups matched to same-ethnicity agents		
	(1)	(2)	(3)	(4)	(5)	(6)
Information treatment	−2.56	−1.65	−1.67	−0.43	−0.33	−0.37
	(1.03)**	(0.97)*	(0.94)*	(0.61)	(0.59)	(0.57)
Low ethnicity agent	−1.91	−0.34	−0.48			
	(1.54)	(1.51)	(1.52)			
Information treatment*Low ethnicity agent	2.49	0.78	0.77			
	(1.21)**	(1.22)	(1.22)			
*R*^2^	0.22	0.24	0.25	0.31	0.33	0.34
*N*	2,106	2,106	2,106	1,395	1,395	1,395
Mean of Control	11.76	11.76	11.76	9.62	9.62	9.62
Agent fixed effects	X	X	X	X	X	X
Controls		Restricted	Full		Restricted	Full

For columns 1–3 the relevant control group is all SHGs that did not receive information and were matched to a high-ethnicity agent. For columns 4–6 the control group is those SHGs that were matched to same ethnicity agents and did not receive information. All regressions include agent fixed effects. We exclude the 117 respondents for whom data on agent name is not available. Restricted controls include respondent age, marital status (1/0), ethnicity (=ST), religion (=Hindu), employment status (1/0), education, HH size, number of children under the age of 5 years, wealth quintiles, total land cultivated, number of years they have been an SHG member and the number of SHG members present at the experiment. Full controls additionally include #food groups consumed, have kitchen garden (1/0), grow vegetables on their land (1/0), agree that produce should be split equally (1/0), dummies for group cohesion and for agent ability. Standard errors (in parentheses) clustered at the SHG level. * *p* < 0.1 ** *p* < 0.05; *** *p* < 0.01.

In the fully specified model of Table 8, as in the corresponding Table 2 above, information continues to be positively and significantly associated with the nutrition knowledge score even when agent fixed effects are included. Similarly, in Table 9, as in Table 3 above, information does not have any association with WTC when the sample is restricted to the set of groups paired with same ethnicity agents. As before, we find that being matched with a low ethnicity agent is negatively associated with WTC, though this is no longer statistically significant. The inclusion of agent fixed effects does appear to attenuate our results, but does not materially change the direction of association.

#### 5.3.2 Perceived ethnicity of agent

We next investigate our assumption that respondents are able to recognize agent ethnicity from their names. As part of our exit survey, we asked respondents to state whether the agent was of the same, higher or lower ethnicity compared to themselves. 59% of the sample correctly identified the ethnicity of the agent. We run the same specifications, but redefine the “treatment” groups based on the respondent’s *perception* of the agent’s ethnicity rather than on the actual ethnicity. For example, the group (H_g_,H_a_, no info) from Fig. 1 is now replaced with (H_g_, H_a_^p^, no info); that is, high-ethnicity respondent matched with agent *perceived* to be of high ethnicity and receiving no information intervention. It follows, therefore, that treatment status may vary within the same experimental group, if for example, some respondents identify the agent as high ethnicity while others perceive her to be low ethnicity. The reader should note that as before, these results cannot be interpreted as being causal, as the sample of women who correctly identified agent ethnicity is clearly non-random. However, the direction of these results relative to the base case presented above will provide additional insights.

We exclude respondents who refused or were unable to answer the ethnic group identification question (19% of the sample), leaving us with 1793 respondents. Of these, 893 thought the agent was of a high ethnicity (low ethnicity groups who said they thought the agent was of higher ethnicity than them AND high ethnicity groups that thought the agent was of the same ethnicity as them) and the rest thought the agent was of a low ethnicity.

Table 10, Table 11 present the results of the same specifications as Table 2, Table 3 above. The results are qualitatively similar both to Table 2, Table 3 and Table 8, Table 9 above. Information continues to be positively and significantly associated with nutrition knowledge score in the full sample and among women in SHGs matched to agents with the same perceived ethnicity as themselves (Table 10). When groups are matched with agents from the perceived low ethnicity group, stated WTC is lower (Table 11, columns 1–3), though the significance of the coefficient is somewhat attenuated with the addition of covariates as compared to the results in Table 3. When restricted to groups matched to same-perceived-ethnicity agents, information is not associated with willingness to contribute (columns 4–6).

**Table 10:** Treatment and nutrition knowledge score, with perceived agent ethnicity.

	Full sample			Groups matched to same-ethnicity agents		
	(1)	(2)	(3)	(4)	(5)	(6)
Information treatment	0.14	0.36	0.40	0.22	0.23	0.25
	(0.12)	(0.11)***	(0.11)***	(0.10)**	(0.09)***	(0.09)***
Low ethnicity agent	−0.26	0.20	0.25			
	(0.15)*	(0.16)	(0.16)			
Information treatment*Low ethnicity agent	0.38	−0.03	−0.07			
	(0.17)**	(0.17)	(0.17)			
*R*^2^	0.01	0.11	0.12	0.00	0.09	0.11
*N*	1,793	1,793	1,793	1,272	1,272	1,272
Mean of Control	7.41	7.41	7.41	7.28	7.28	7.28
Controls		Restricted	Full		Restricted	Full

For columns 1–3 the relevant control group is those SHGs that did not receive information and were matched to an agent perceived to be of high-ethnicity. For columns 4–6 it is those SHGs that were matched to agents perceived to be of the same ethnicity and who did not receive information. Restricted controls include respondent age, marital status (1/0), ethnicity (=ST), religion (=Hindu), employment status (1/0), education, HH size, number of children under the age of 5 years, wealth quintiles, total land cultivated, number of years they have been an SHG member and the number of SHG members present at the experiment. Full controls additionally include #food groups consumed, have kitchen garden (1/0), grow vegetables on their land (1/0), agree that produce should be split equally (1/0), dummies for group cohesion and for agent ability. Standard errors (in parentheses) clustered at the SHG level. * *p* < 0.1 ** *p* < 0.05; *** *p* < 0.01.

**Table 11:** Treatment and willingness to contribute labor hours, with perceived agent ethnicity.

	Full sample			Groups matched to same-ethnicity agents		
	(1)	(2)	(3)	(4)	(5)	(6)
Information treatment	−2.00	−1.75	−1.62	−0.33	−0.34	−0.28
	(1.20)*	(1.22)	(1.16)	(0.84)	(0.78)	(0.75)
Low ethnicity agent	−4.10	−3.70	−3.35			
	(1.22)***	(1.37)***	(1.30)**			
Information treatment*Low ethnicity agent	2.37	1.98	1.59			
	(1.36)*	(1.46)	(1.40)			
*R*^2^	0.04	0.06	0.10	0.00	0.08	0.12
*N*	1,793	1,793	1,793	1,272	1,272	1,272
Mean of Control	11.70	11.70	11.70	9.63	9.63	9.63
Controls		Restricted	Full		Restricted	Full

For columns 1–3 the relevant control group is those SHGs that did not receive information and were matched to an agent perceived to be of high-ethnicity. For columns 4–6 it is those SHGs that were matched to agents perceived to be of the same ethnicity and who did not receive information. Restricted controls include respondent age, marital status (1/0), ethnicity (=ST), religion (=Hindu), employment status (1/0), education, HH size, number of children under the age of 5 years, wealth quintiles, total land cultivated, number of years they have been an SHG member and the number of SHG members present at the experiment. Full controls additionally include #food groups consumed, have kitchen garden (1/0), grow vegetables on their land (1/0), agree that produce should be split equally (1/0), dummies for group cohesion and for agent ability. Standard errors (in parentheses) clustered at the SHG level. * *p* < 0.1 ** *p* < 0.05; *** *p* < 0.01.

#### 5.3.3 The impact of the treatment on perceived agent ability and group cohesion

In our main results, we found that the information treatment was associated with an increased nutrition knowledge score but that this did not translate into increased WTC even among groups matched to same-ethnicity agents. On the other hand, being matched to a low-ethnicity agent appears to be negatively associated with the WTC regardless of the ethnicity of the group, overall, and regardless of information provision.

We mention above that these results might be due to the novelty of the low ethnicity agent providing information, that drives up information retention, combined with the perceived inability of the same agent to actually facilitate the construction of the kitchen garden, that drives down WTC. In addition, through the priming of ethnic identity, the ethnicity treatment might also change the respondent’s perceptions about her own group identity and the level of intra-group cooperation or cohesiveness. To check these, we look at the associations of both the information and ethnicity treatments with the perceived ability of the agent and the individual reports of group cohesiveness. Ethnicity-related biases could affect an individual’s assessment of agent ability, for example higher-ethnicity groups being dismissive of lower-ethnicity agents. Similarly, being paired with an agent that is of shared or different ethnicity might cause respondents to alter their perception of their group’s cohesion. The raw distributions of these two variables are provided in Figs. A2.2 and A2.3.

Table A2.3 presents results using perceived agent ability and group cohesion as dependent variables. Panel A of the table uses the respondent’s perception of agent ability as the dependent variable, while Panel B uses the respondent’s perception of group cohesion. Within each panel, the first two columns use the full sample and the next two the set of groups matched to same ethnicity agents.

Interestingly, in Panel A, the information treatment has a positive and weakly significant association with perceived agent ability in the full sample, but not in the sample of groups matched to same ethnicity agents. For the full sample, in the model with covariates (Column 2) this coefficient is 0.15, or about 2.8% of the control arm mean. For the set of groups matched to same ethnicity agents, the association is smaller at 0.07, or approximately 1.3% of the control arm mean and is not significant. The ethnicity treatment does not affect respondent perception of agent ability, however providing information to the group is associated with positive perception of agent capabilities.

In Panel B, we don’t find any relationship between either the information or ethnicity treatments and perceived group cohesion. This suggests that the results we noted above on the WTC are not driven by an increase in the respondent’s sense of group solidarity that might drive them to be any more or less cooperative than otherwise.

### 5.4 Other covariates

The full specifications of the regression equations discussed above also exhibit some associations of interest with individual-, household- and group-level characteristics (see Appendix tables A2.4 and A2.5). These are not impacts, but mediators, and in this section we discuss those mediators that can arguably be treated as being exogenous to the treatment.

Older women have lower scores on the knowledge test (Table A2.4), but age does not have any association with WTC (Table A2.5). Relative to the category of no education, greater educational attainment is associated with higher scores on the knowledge test. This is to be expected; more educated individuals are both more likely to have access to similar information from elsewhere, but also to be able to retain information more accurately. We see a similar positive education gradient with respect to WTC, but this is not statistically significant. Wealth quintiles, based on asset ownership, are not associated with WTC, but are associated with knowledge scores, with the richest two quintiles demonstrating significantly higher scores than the poorest base category. Wealth is likely to be correlated with both education and ethnicity.

One might argue that the longer women are part of the group, the more likely they are to be cooperative. While on average, OBC members have been a part of the group marginally longer than ST members, we find no relationship between length of SHG duration and knowledge score or WTC. Fig. A2.4 shows the distribution of WTC by duration of SHG membership for low and high ethnicity groups, and we see no notable pattern in the relationship between membership duration and WTC for either of the two groups.

Finally, women who currently had a home garden scored higher on the nutrition knowledge test (Table A2.4), perhaps because the information being provided had greater salience for them. Having a home garden was also positively and significantly correlated with WTC. However, once again the connection between retaining information and WTC appears tenuous: women with higher self-reported dietary diversity volunteered a significantly greater number of labor hours (Table A2.5), even though they did not have higher knowledge score.

## 6 Discussion and policy relevance

Using a multi-arm randomized controlled trial conducted with women’s SHGs in the eastern Indian state of West Bengal, we study how providing information in group settings is associated with individual knowledge about nutrition and willingness to contribute labor hours towards the construction and maintenance of a group-owned kitchen garden. More importantly, we also test how information provision is moderated by a shared ethnic identity with the agent providing the information. To do this, we cross-randomize two treatments – the *information treatment*, i.e., whether the group receives any nutrition information motivating the need for a kitchen garden, and the *ethnicity treatment*, i.e., the ethnic identity of the agent relative to the identity of the group. Our cross-randomization allows us to identify the relationship between information and shared ethnic identities, with willingness to contribute and knowledge retention. Since we were unable to fully cross-randomize, we are not able to make causal claims for the full sample, but associations in the full sample and in the subset of groups that received the information treatment reveal the salience of ethnicity in driving respondent behavior. Our experimental design accounts for incentive compatibility constraints and other location- and group-specific constraints documented during our qualitative fieldwork and scoping visits.

We find that providing information is associated with a significant increase in the respondent’s knowledge score, however the extent of this relationship depends on the ethnicity of the agent relative to the group. When groups are matched with low- ethnicity agents, information provision is positively associated with knowledge retention, indicating that information from low- ethnicity agents may be retained better. This could be because of the novelty of a low ethnicity agent commanding center-stage and delivering information, causing women to pay more attention. When restricted to the subsample of same- ethnicity agents and groups, eliminating the possible confounding impacts of shared identity, the relationship between receiving nutrition information and knowledge score is still statistically significant.

We find no relationship between shared identity and WTC, indicating that being matched to a same- ethnicity agent does not inherently change one’s valuation of the public good or WTC. But ethnicity does matter. Being matched to a low- ethnicity agent is associated with a significantly lower individual willingness to contribute labor hours, despite the fact that it is associated with increased score on the knowledge test. This relationship seems to be driven by high-group respondents matched with low-ethnicity agents, and could be due to these individuals’ limited confidence in the ability of the low- ethnicity agent to provide the public good. High-ethnicity agents may be perceived to have greater access to the resources needed to deliver services, encouraging individuals to volunteer a higher number of labor hours despite lower retention of information.

Our focus on possible mechanisms is largely from a group member perspective. However, there are also likely to be agent-specific factors that influence how effective the agent is and how she is perceived by the group. For example, a lower-ethnicity agent might feel intimidated addressing a higher-ethnicity group, and this could translate into the message being less well-received or the agent’s ability being underestimated by group members. While we are unable to fully test this, there are several reasons why we believe this is not a large concern. First, we show that agents of different ethnicities do not differ greatly on observable characteristics. Second, we include agent-fixed effects, and perceived agent identity, in our analysis – while this does not account for differences in agent behavior across high- and low-ethnicity groups, it does allow us to account for unobservable agent characteristics such as confidence, experience in leadership or public speaking, and comfort in addressing large groups. Doing so only attenuates the results slightly, and our main messages still stand. Third, we took considerable care to train agents on the scripted content of the information session and to engage them in role-play simulating the group meetings. At the end of the training session, agents were able to recite the content from memory, limiting differences across agents as well as within an agent across groups. Finally, and most importantly, if agents do indeed act differently depending on the ethnicity of the group they are interacting with, this should be considered an inherent part of what ethnic identity in a hierarchical social system entails. Thus, our conclusions continue to be relevant, particularly in scenarios where possible agent discomfort in addressing a group of a different ethnicity is a very real part of everyday social equations.

Our findings have important implications for the design and implementation of programs that rely on groups as vehicles of change, particularly in societies that are ethnically heterogenous and socially stratified. We find that group cohesion, while not associated with knowledge retention, is positively associated with an individual’s willingness to contribute. Engaging groups in activities that build trust and cohesion may thus be a critical first step to increasing and sustaining participation in collective action initiatives (Sabhlok, 2011, Hoff and Pandey, 2005). This may also mean that less mature or less cohesive groups may be less able to effect change and hence unprepared to act as vehicles for NGO- or government-led interventions (Walker et al., 2010). Respondents paired with agents whom they perceived to be more articulate and confident scored significantly higher on the knowledge test. This means that, in addition to extensive training, interventions need to invest in the development of soft skills. Programs and interventions would do well to follow a capacity building model so that they equip their field agents with the skills needed to effectively communicate with and reach out to a diverse group of beneficiaries with whom they may or may not share a common social identity.

Both the quantitative data and qualitative evidence from the field indicate that the opportunity cost of time varies widely for different caste groups (Eswaran et al., 2013), mediating their ability to participate in group-based interventions and activities that require time and effort. Interventions that advocate the adoption of time-intensive practices, as in the maintenance of kitchen gardens, should expect different ethnic groups to respond differently to these interventions. This should also be factored into the design of these programs.

Although we tested the effect of ethnicity and identity using only a one-time information intervention and a knowledge test administered immediately after the information was provided, the long-term retention of information, how groups act on this information, and possible spillover effects on other behaviors, are open questions that deserve further research. As we noted in the design section, our experiment cannot distinguish between effect of shared identity with the agent, and effects of being paired with a low ethnicity agent in the case of low ethnicity group or a high ethnicity agent in the case of a high ethnicity group. Doing so will require replicating this experiment with a larger set of ethnicities; this remains an open empirical question and one that we hope to address in future work.

Our results are consistent with the global evidence that nutrition-sensitive interventions are more successful when combined with social and behavior change communication (Warren et al., 2020, SPRING, 2018), a fact that is increasingly being acknowledged in the Indian policy landscape as well. It is noteworthy that SBCC is a core intervention component of the National Nutrition Mission (NITI Aayog, 2018), making our results policy relevant. The larger implications of our work, however, pertain to the design of service delivery systems. In developing country settings, agriculture, health and nutrition information, and other services are provided through agents who belong to the communities they serve. These communities need not be homogenous in ethnic or social identity. It is crucial to understand how social identity and other divisions can bolster or undermine otherwise well-designed interventions to effectively allocate agents among communities or provide additional support to overcome possible barriers. The policy of closely matching agent ethnicity to that of the beneficiaries they serve is one that many grassroots organizations with a strong local presence and a deep understanding of the local context have adopted. For example, PRADAN now routinely employs agents from tribal groups who work in tribal-dominated areas, and the same for other ethnic identities. For more centrally supported and administered programs, this design feature is yet to be fully recognized and appreciated. We hope that this research informs the design and implementation of frontline agent-led development programs going forward.

## CRediT authorship contribution statement

**Kalyani Raghunathan:** Conceptualization, Methodology, Validation, Formal analysis, Writing – original draft, Visualization, Funding acquisition, Writing – review & editing, Project administration, Supervision. **Muzna Alvi:** Conceptualization, Methodology, Validation, Formal analysis, Writing – original draft, Visualization, Funding acquisition, Writing – review & editing. **Mrignyani Sehgal:** Methodology, Validation, Formal analysis, Writing – original draft.

## Declaration of Competing Interest

The authors declare that they have no known competing financial interests or personal relationships that could have appeared to influence the work reported in this paper.

## Footnotes

1 It is relatively well-established that high caste agents are overrepresented among all rungs of government, judiciary, and law enforcement. See for example https://www.scobserver.in/journal/caste-diversity-at-the-supreme-court/ and https://www.barandbench.com/columns/disproportionate-representation-supreme-court-caste-and-religion-of-judges on the lack of diversity in the Supreme Court of India, and Verma (2019) on the lack of representation in politics more broadly. On frontline worker characteristics, a study in Madhya Pradesh found that Anganwadi workers were predominantly from the General castes (44%), followed by OBCs and STs at around a quarter, with SCs trailing at only 5% (see Avula et al., 2015a). A similar study in Odisha found that 52.2% of the Anganwadi workers were OBC, with only 18% being ST (see Avula et al., 2015b).

2 In blocks where PRADAN is operational, other governmental or non-governmental groups are not involved in organizing women into SHGs. Thus, the list obtained from PRADAN forms the universe of SHGs in this area. SHGs need to be defunct for six months to be considered dissolved – this means some of the SHGs in the list may not have existed at the time of the survey if they had only recently ceased to meet, however, barring issues of omission of this nature, there is no indication that there is a clear reporting bias of any kind in the monitoring data.

3 These wealth quintiles were calculated using principal component analysis on the full set of assets owned.
