# Supplementary material for: Ethnicity, information and cooperation: Evidence from a group-based nutrition intervention
Source: Food Policy. 2023 Oct;120:102478. doi: 10.1016/j.foodpol.2023.102478 (PMC10679797; doi:10.1016/j.foodpol.2023.102478)
Supplement: Supplementary data 1 [file mmc1.docx]

**Ethnicity, Information and Cooperation: Evidence from a group-based nutrition intervention**

Supplementary Online Appendix

# Appendix A1: Scripted sessions

**A1.1: Script of session for the “information” treatment group**

Namaste to all the didis! My name is _________. I am from ________village.

Today we are going to talk about food habits- food you eat and grow that can make you and your family healthier.

I will begin with some information on the importance of a good diet. You might have heard that it is important to eat the right types of foods and in the right amounts so that your body is provided with the nutrients it needs for its functioning. What people should eat and how much of it they should eat changes with age. However, the three basic rules of eating are the same for all ages.

*First:* it is very important that the food we eat provides us with all the nutrients we need. The body needs nutrients to be able to grow and develop, to fight against illness, to provide energy for the activities you have to carry out during the day. Different foods contain different types of nutrients. The best way to ensure that you receive all the nutrients you need is to eat several different kinds of foods.

*Second:* Different individuals will need different amounts of food based on their age, sex, level of physical activity, or health status. Pregnant and lactating mothers, for example, need to eat more than they usually would.

*Third:* Meals should be prepared in a safe and hygienic environment, to keep the food germ free and suitable for consumption.

***

**Question to the group:** Now tell me, what did you all eat yesterday? I would like to hear from each of you about what you ate yesterday from morning till night. Please also tell us where did you get the food you ate? (Did you buy it, did you grow it in your fields or near your home, or did someone give it to you?)

Now that we have learnt the basic principles of healthy eating, and we have heard from all of you about what kinds of foods you eat in a normal day, let’s talk a bit more about the different types of foods and their functions.

Some of you might already know that foods can be divided into three basic food groups. Each group of foods performs a different function and is important for the body in a different way. I will tell you a little about each of these groups. During this time, listen carefully so that afterwards, you can give me some examples of foods of each group.

1. The first group of foods are the *energy giving foods*: The foods that give your body the energy it needs, for example, to work on the farms, for children to play, for household chores. These are the foods that provide your body with the energy it needs to move and be active, to keep organs like the heart and lungs functioning, and so on.

**Question to the group**: What are some examples of these types of foods?

[allow women to discuss but don’t offer any answers or comments]

**Answer**: Some examples of these foods would be all grains, like rice, roti, bread, corn; sugar, honey, jaggery; oil or ghee; potato and sweet potato and so on.

1. The second group of foods are the *body building foods*: Foods that help your body grow and repair itself, e.g. to build and maintain muscle, blood, bones. Some of these are the foods that come from animal sources. But there are also other types of foods that help the body grow.

**Question to the group**: What are some examples of these types of foods?

[allow women to discuss but don’t offer any answers or comments]

**Answer**: All types of pulses – *moong, masoor, arhar/raher, kurthi, khesari, kurthi, kolai, chana, rajma*. Soyabean is also a great source. Other animal source foods include milk and milk products, like *dahi, paneer*; eggs; mutton; chicken; fish, duck

1. The third group of foods are the *protective foods*: Foods that protect your body from illness and help prevent you from falling sick. These foods provide the micronutrients and vitamins that keep you healthy. If your diet does not include enough of these foods, you will find yourself falling sick again and again.

**Question to the group**: What are some examples of these types of foods?

[allow women to discuss but don’t offer any answers or comments]

**Answer**: Green leafy vegetables, like *saag, palak, methi, kolmi saag, pui saag*. Fruits, like papaya, orange, guava, grapes, apples, mango.

It is very important that your body receives a mixture of these foods and the nutrients they provide with every meal. The only way to do this is to try your best to eat some foods from each group with every meal.

***

Undernutrition occurs when your body doesn’t get enough of the nutrients it should get to function properly and in a healthy manner. The problem of undernutrition is common in rural areas, especially among women and young children. There are several reasons for undernutrition in these groups, one of them is lack of knowledge about the right foods to eat, another is the lack of resources needed to purchase the kinds of foods one needs to eat.

Most of you mention that you ate roti/rice yesterday. Many of you also mentioned eating daal. These two foods belong to the *energy giving* and *body building* food groups. But it is very important to also eat fruits and vegetables, the *protective* foods. Not only do they provide important vitamins and minerals and protect us from illness, they also help in digesting our food better. These foods are particularly important for pregnant and lactating women, and for young children.

For example, not having enough iron-rich foods can lead to anaemia- which can cause severe fatigue and tiredness in adults and children. One way to prevent and treat anaemia is to consume green leafy vegetables that are rich in iron, such as spinach, *saag, sarson, pui saag, note saag, methi, kul saag*, etc.

Another example is, not eating foods rich in vitamin A can cause night blindness (reduce ability to see in the dark). Along with dairy products and green leafy vegetables, one great source of Vitamin A is carrots.

[CDC facilitator reminds the group of some of the places where they get fruits and vegetables from]

One easy way of making sure that you have enough of protective foods in your diet is to grow fruits and vegetables around your home. This way you can also save money as you will not need to buy the same foods on the market. At the same time, you and your families will be healthier as a result of eating these foods.

Some of you said that you do grow foods around your home, or that you foraged for foods from the forest. It is easy to grow fruit trees like papaya, mango, pomegranate, dates, jackfruit, guava, ber and jamun. Vegetables like pumpkin and lauki can also be easily grown on the roof of your house.

If you want to grow enough fruits and vegetables to feed your whole family, however, you will need to set aside a larger space. One way of doing this is to set up a kitchen garden. In this kitchen garden, you can grow several types of fruits and vegetables at the same time, and all year round, so that you always have something to eat without depending on the market!

For example, during the time of kharif, a lot of vegetables are not available in the market. During this time, you can grow vegetables like lauki in your kitchen garden.

I know that you may not have the time to maintain a large kitchen garden by yourself. However, just like you all pool small amounts of money to create a large sum for all the group members, you can lease and operate a group kitchen garden where you all share in the labor and output. As a group you can collectively lease a plot of land, and on this land, you can grow all kinds of fruits and vegetables.

Other than a small investment to procure seeds and saplings, there is no monetary payment required to participate in this kitchen garden. Instead of money, you all can invest hours of your own time to maintain and grow the garden. In addition to growing vegetables in the garden, you can also use the garden to grow saplings in the nursery that you can then take and transplant in your own private garden. As a group you can decide how to split the share of the produce- you can share it based on each member’s needs, based on the hours of work put in by each didi, or the output can be split equally.

Let us imagine that it is possible to build such a garden together with your group-I want you to think about how many hours you would be willing to contribute per week to maintain the garden.

The total hours of labor that will be put into the kitchen garden will be a sum of the total hours every member of the group contributes. For example, if there are 3 members in the group and one member contributes 7, another member contributes 10 and the third member contributes 3 hours, then the total time invested in taking care of the garden is 7+10+3=20 hours per week.

We are asking this question to many other self-help groups in this block. In a few groups selected through lottery we will help in construction of a community kitchen garden in real life.

Our help will be in the form of advice on how to construct the garden, what types of vegetables to grow, how to maintain distance between crops etc. Thus, make sure you reveal the true number of hours you are willing to contribute, because there is a chance your group may be selected for construction of a group kitchen garden.

Suppose you worked 8 hours a day for a whole week. The maximum you could work on the garden is 56 hours. Each of you has one red bag with 56 beads and each bead represents one hour. I want you to first decide how many hours of work and labor you are willing to contribute to this group/community kitchen garden **PER WEEK**. Please don’t discuss this with other members of the group. Your level of contribution can be higher or lower than that of others in your group. You can also choose to invest zero or no time to this garden.

For every hour you are willing to put in, take the equivalent number of beads and put it in the blue bag in front of you. For example, if you are willing to work for 7 hours a week, take 7 beads from the red bag and put it in the blue bag. Similarly, if you are willing to put in 12 hours a week, put 12 beads in the bag. Remember, you **are allowed to invest “zero”** hours, in which case you would put no beads in the blue bag.

Make sure no one else but you can see the number of beads you put in the bag. Only we will be able to see how many hours of labour you volunteer to contribute- no one else in your group will know the number of hours you decide and the number of beads you put in the blue bag- either now or anytime in the future.

Once you have placed the beads in the bag keep the bag with you and proceed outside where we have some people who would like you to answer a small survey about yourself and about your cultivation practices. Remember not to discuss the number of beads or the number of hours you have volunteered to contribute to the group kitchen garden with other members of the group until you have answered the survey questions.

**A1.2: Script of session for the “no-information” treatment group**

Namaste to all the didis! My name is _________. I am from ________village.

Today I will talk to you about kitchen gardens. Many of you may have already heard about them. Some of you may already have a kitchen garden in your home. However, what if instead of a small private kitchen garden, you could cultivate a large kitchen garden?

I know that you may not have the time to maintain a large kitchen garden by yourself. However, just like you all pool small amounts of money to create a large sum for all the group members, you can lease and operate a group kitchen garden where you all share in the labor and output. As a group you can collectively lease a plot of land, and on this land, you can grow all kinds of fruits and vegetables.

Other than a small investment to procure seeds and saplings, there is no monetary payment required to participate in this kitchen garden. Instead of money, you all can invest hours of your own time to maintain and grow the garden. In addition to growing vegetables in the garden, you can also use the garden to grow saplings in the nursery that you can then take and transplant in your own private garden. As a group you can decide how to split the share of the produce- you can share it based on each member’s needs, based on the hours of work put in by each didi, or the output can be split equally.

Let us imagine that it is possible to build such a garden together with your group-I want you to think about how many hours you would be willing to contribute per week to maintain the garden.

The total hours of labor that will be put into the kitchen garden will be a sum of the total hours every member of the group contributes. For example, if there are 3 members in the group and one member contributes 7, another member contributes 10 and the third member contributes 3 hours, then the total time invested in taking care of the garden is 7+10+3=20 hours per week.

We are asking this question to many other self-help groups in this block. In a few groups selected through lottery we will help in construction of a community kitchen garden in real life.

Our help will be in the form of advice on how to construct the garden, what types of vegetables to grow, how to maintain distance between crops etc. Thus, make sure you reveal the true number of hours you are willing to contribute, because there is a chance your group may be selected for construction of a group kitchen garden.

Suppose you worked 8 hours a day for a whole week. The maximum you could work on the garden is 56 hours. Each of you has one red bag with 56 beads and each bead represents one hour. I want you to first decide how many hours of work and labor you are willing to contribute to this group/community kitchen garden **PER WEEK**. Please don’t discuss this with other members of the group. Your level of contribution can be higher or lower than that of others in your group. You can also choose to invest zero or no time to this garden.

For every hour you are willing to put in, take the equivalent number of beads and put it in the blue bag in front of you. For example, if you are willing to work for 7 hours a week, take 7 beads from the red bag and put it in the blue bag. Similarly, if you are willing to put in 12 hours a week, put 12 beads in the bag. Remember, you **are allowed to invest “zero”** hours, in which case you would put no beads in the blue bag.

Make sure no one else but you can see the number of beads you put in the bag. Only we will be able to see how many hours of labour you volunteer to contribute- no one else in your group will know the number of hours you decide and the number of beads you put in the blue bag- either now or anytime in the future.

Once you have placed the beads in the bag keep the bag with you and proceed outside where we have some people who would like you to answer a small survey about yourself and about your cultivation practices. Remember not to discuss the number of beads or the number of hours you have volunteered to contribute to the group kitchen garden with other members of the group until you have answered the survey questions.

# Appendix A2: Figures and Tables
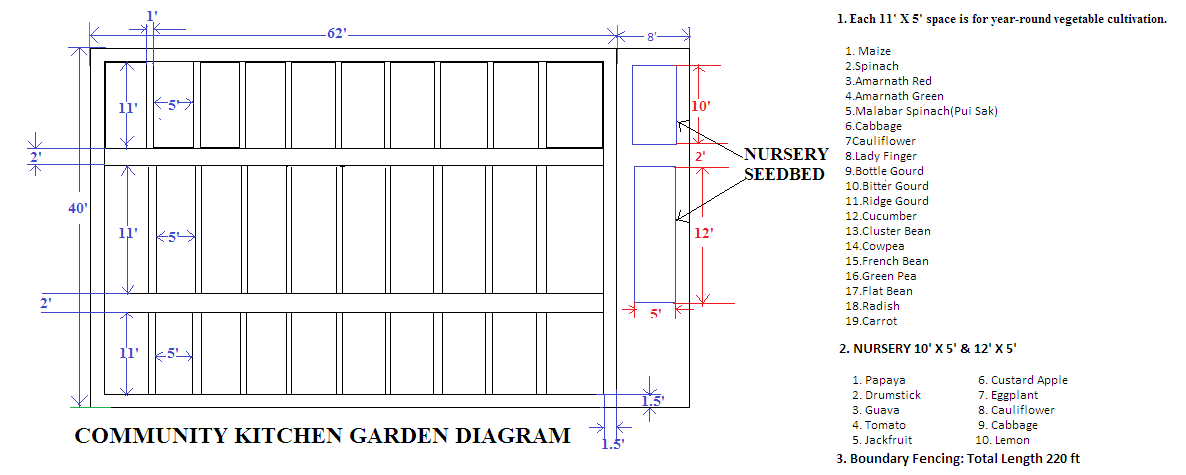


**Figure A2.1: Kitchen garden model developed by PRADAN**


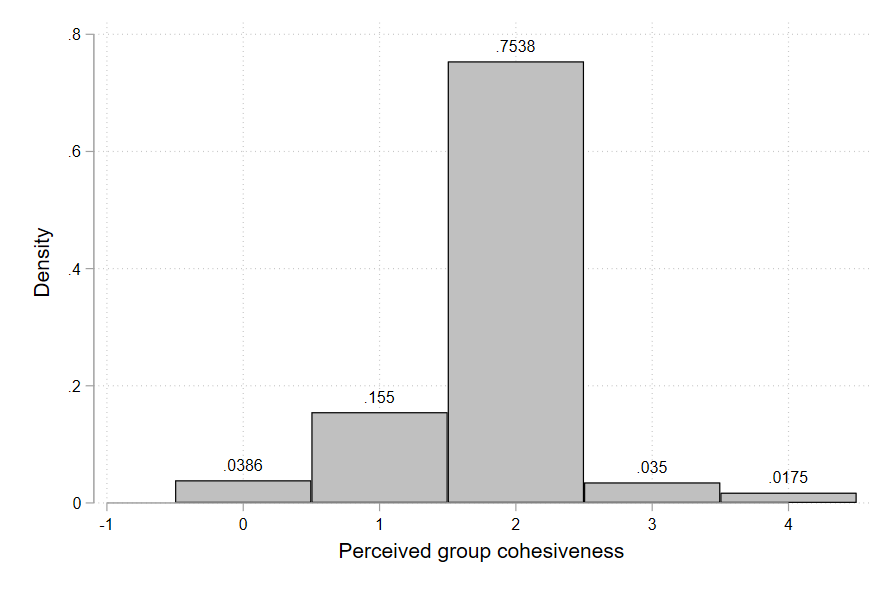


**Figure A2.2: Measure of perceived group cohesiveness, sum of scores on four yes/no questions**


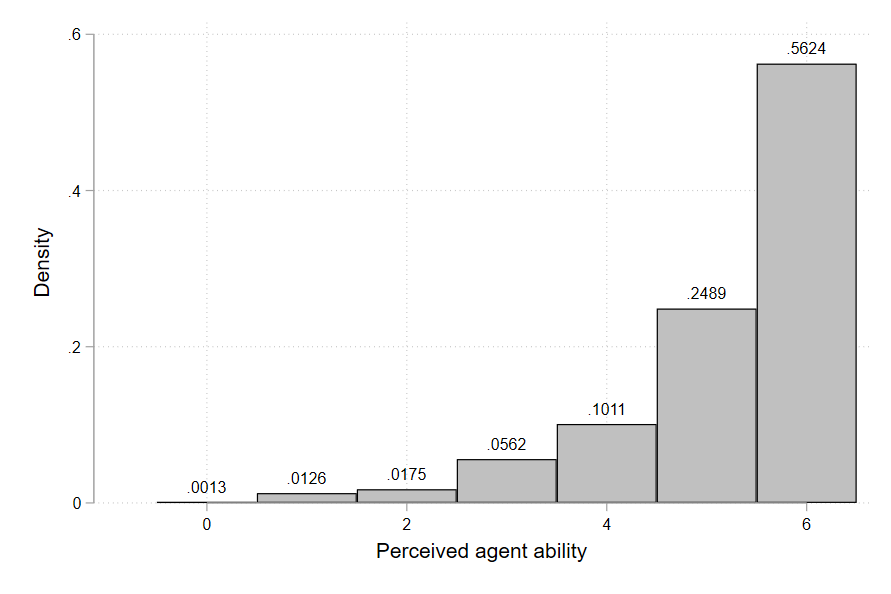


**Figure A2.3: Measure of perceived agent ability, sum of scores on six yes/no questions**

**Figure A2.4: Mean willingness to contribute labor hours, by duration of SHG membership**

**Table A2.1: Descriptive statistics from NFHS-4 (2015-16), disaggregated by ethnic groups- West Bengal**

|  | **ST** | | **SC** | | **OBC** | | **General** | | **Tests for difference (p values)** | | |
| --- | --- | --- | --- | --- | --- | --- | --- | --- | --- | --- | --- |
|  | **Mean (SD)/Propn** | **N** | **Mean (SD)/Propn** | **N** | **Mean (SD)/Propn** | **N** | **Mean (SD)/Propn** | **N** | **ST vs SC** | **ST vs OBC** | **ST vs General** |
| Respondent woman education (years) | 4.21(4.3) | 1373 | 5.26(4.4) | 5203 | 6.97(4.4) | 2208 | 7.23(4.5) | 5615 | 0 | 0 | 0 |
| Age of respondent at 1st birth | 19.56(3.8) | 998 | 18.88(3.3) | 3942 | 19.3(3.4) | 1584 | 19.73(3.8) | 4230 | 0 | 0.22 | 0.52 |
| Total number of children ever born | 1.7(1.5) | 1373 | 1.74(1.4) | 5203 | 1.72(1.6) | 2208 | 1.65(1.4) | 5615 | 0.64 | 0.87 | 0.45 |
| Husband's educational attainment (years) | 1.72(1.6) | 207 | 1.94(1.6) | 659 | 2.4(1.5) | 282 | 2.46(1.6) | 795 | 0.41 | 0.02 | 0.01 |
| Body mass index | 20.2314(3.84) | 1350 | 21.1143(3.646) | 5120 | 21.6516(4.289) | 2178 | 22.1925(4.455) | 5452 | 0 | 0 | 0 |
| Wealth index: HH belongs to __ quintile: |  |  |  |  |  |  |  |  |  |  |  |
| poorest | 46.8 | 1373 | 27.2 | 5203 | 13.6 | 2208 | 12.7 | 5615 | 0.005 | 0 | 0 |
| poorer | 23.7 | 1373 | 24.2 | 5203 | 21.3 | 2208 | 16.5 | 5615 | 0.886 | 0.489 | 0.064 |
| middle | 14.0 | 1373 | 21.8 | 5203 | 23.3 | 2208 | 21.1 | 5615 | 0.023 | 0.005 | 0.091 |
| richer | 11.1 | 1373 | 17.1 | 5203 | 25.1 | 2208 | 23.6 | 5615 | 0.059 | 0.002 | 0.004 |
| richest | 4.4 | 1373 | 9.7 | 5203 | 16.7 | 2208 | 26.1 | 5615 | 0 | 0 | 0 |
| HH has electricity | 83.3 | 1373 | 90.1 | 5203 | 92.6 | 2208 | 92.8 | 5615 | 0.001 | 0.001 | 0 |

Source: Authors’ calculations using the district-representative National Family Health Survey (NFHS) round 4, 2015-16.

**Table A2.2: Agent characteristics by ethnicity**

|  | ST | OBC | Total |
| --- | --- | --- | --- |
| Variable | Mean (SE) | Mean (SE) | Mean (SE) |
|  | (1) | (2) | (3) |
| Age in years | 26.00 | 30.40 | 28.44 |
|  | (2.35) | (1.50) | (1.46) |
| Years of education | 12.25 | 10.60 | 11.33 |
|  | (1.03) | (1.33) | (0.87) |
| Number of assets | 10.75 | 13.80 | 12.44 |
|  | (1.25) | (0.97) | (0.90) |
| Number of children | 1.50 | 2.00 | 1.78 |
|  | (0.50) | (0.00) | (0.22) |
| Number of years as CDC | 4.25 | 3.70 | 3.94 |
|  | (0.85) | (0.80) | (0.56) |
| No. of years as SHG member | 6.50 | 7.60 | 7.11 |
|  | (0.96) | (1.83) | (1.06) |
| Height (in cm) | 144.20 | 152.60 | 148.87 |
|  | (3.32) | (2.36) | (2.36) |
| Agree that people of own ethnicity pay more attention | 0.75 | 0.00 | 0.33 |
|  | (0.25) | (0.00) | (0.17) |
| Agree that people of higher ethnic group pay more attention | 0.75 | 0.20 | 0.44 |
|  | (0.25) | (0.20) | (0.18) |
| Self-reported score on self esteem (range 1-4) | 2.50 | 3.60 | 3.11 |
|  | (0.65) | (0.40) | (0.39) |
| Self-reported score on locus of control (range 1-4) | 2.00 | 2.00 | 2.00 |
|  | (0.00) | (0.00) | (0.00) |
| Self-reported score on social desirability | 9.25 | 11.20 | 10.33 |
|  | (0.48) | (0.58) | (0.50) |
| Self-reported score on motivation (range 1-7) | 6.75 | 6.40 | 6.56 |
|  | (0.25) | (0.24) | (0.18) |
| Self-reported score on self confidence (range 1-4) | 4.00 | 4.00 | 4.00 |
|  | (0.00) | (0.00) | (0.00) |
| N | 4 | 5 | 9 |
| Notes: Authors’ calculations. Standard errors in parentheses. |  |  |  |

**Table A2.3: Association of information and ethnicity treatments with perceived agent ability and group cohesion**

|  | Full sample | | | Groups matched to same-ethnicity agent | | |
| --- | --- | --- | --- | --- | --- | --- |
|  | (1) | | (2) | (3) | | (4) |
| Panel A: Perceived agent ability | | | | | | |
| Information treatment | 0.08 | 0.15 | | 0.07 | 0.07 | |
|  | (0.08) | (0.08)* | | (0.07) | (0.07) | |
| Low ethnic group agent | -0.08 | 0.08 | |  |  | |
|  | (0.09) | (0.11) | |  |  | |
| Low ethnic group agent x Information treatment | -0.02 | -0.18 | |  |  | |
|  | (0.11) | (0.14) | |  |  | |
| *R*^2^ | 0.00 | 0.02 | | 0.00 | 0.02 | |
| *N* | 2,223 | 2,223 | | 1,465 | 1,465 | |
| Mean of Control | 5.23 | 5.23 | | 5.19 | 5.19 | |
| Controls |  | Restricted | |  | Restricted | |
| Panel B: Perceived group cohesion | | | | | | |
| Information treatment | -0.01 | -0.00 | | -0.04 | -0.04 | |
|  | (0.05) | (0.05) | | (0.04) | (0.04) | |
| Low ethnic group agent | -0.02 | 0.00 | |  |  | |
|  | (0.06) | (0.07) | |  |  | |
| Low ethnic group agent x Information treatment | -0.02 | -0.04 | |  |  | |
|  | (0.07) | (0.08) | |  |  | |
| *R*^2^ | 0.00 | 0.01 | | 0.00 | 0.01 | |
| *N* | 2,223 | 2,223 | | 1,465 | 1,465 | |
| Mean of Control | 1.86 | 1.86 | | 1.86 | 1.86 | |
| Controls |  | Restricted | |  | Restricted | |
| For columns 1-2 the relevant control group is those SHGs that did not receive information and were matched to an agent of high-ethnic group. For columns 3-4 it is SHGs that did not receive information and were matched to agents of same ethnic group. Restricted controls include respondent age, marital status (1/0), ethnicity (=ST), religion (=Hindu), employment status (1/0), education, HH size, number of children under the age of 5 years, wealth quintiles, total land cultivated, number of years they have been an SHG member and the number of SHG members present at the experiment. Standard errors (in parentheses) clustered at the SHG level. * *p*<0.1 ** *p*<0.05; *** *p*<0.01 | | | | | | |

**Table A2.4: Association of the information and ethnicity treatments with the nutrition knowledge score (full estimation)**

|  | Full sample | | | Groups matched to same-ethnicity agents | | |
| --- | --- | --- | --- | --- | --- | --- |
| Information treatment | -0.05 | 0.21 | 0.20 | 0.39 | 0.40 | 0.40 |
|  | (0.12) | (0.10)** | (0.10)** | (0.09)*** | (0.08)*** | (0.08)*** |
| Low ethnic group agent | -0.15 | 0.33 | 0.35 |  |  |  |
|  | (0.14) | (0.15)** | (0.15)** |  |  |  |
| Information treatment*Low ethnic group agent | 0.75 | 0.28 | 0.30 |  |  |  |
|  | (0.17)*** | (0.16)* | (0.16)* |  |  |  |
| Age |  | -0.02 | -0.02 |  | -0.01 | -0.01 |
|  |  | (0.00)*** | (0.00)*** |  | (0.00)*** | (0.00)*** |
| Married |  | 0.05 | 0.03 |  | -0.06 | -0.10 |
|  |  | (0.11) | (0.11) |  | (0.13) | (0.14) |
| Scheduled Tribe |  | -0.27 | -0.26 |  | 0.19 | 0.24 |
|  |  | (0.08)*** | (0.09)*** |  | (0.09)** | (0.10)** |
| Religion: Hindu |  | -0.03 | -0.08 |  | -0.18 | -0.23 |
|  |  | (0.14) | (0.14) |  | (0.12) | (0.13)* |
| Respondent woman is employed |  | 0.17 | 0.15 |  | 0.11 | 0.10 |
|  |  | (0.09)* | (0.09)* |  | (0.10) | (0.11) |
| Completed less than grade 5 |  | 0.23 | 0.24 |  | 0.28 | 0.26 |
|  |  | (0.10)** | (0.10)** |  | (0.12)** | (0.12)** |
| Completed between grades 5 and 8 |  | 0.49 | 0.52 |  | 0.61 | 0.60 |
|  |  | (0.12)*** | (0.13)*** |  | (0.17)*** | (0.17)*** |
| Completed grade 9 or higher |  | 0.54 | 0.58 |  | 0.58 | 0.59 |
|  |  | (0.13)*** | (0.13)*** |  | (0.17)*** | (0.17)*** |
| Household size |  | 0.00 | 0.01 |  | -0.00 | 0.00 |
|  |  | (0.02) | (0.02) |  | (0.02) | (0.02) |
| # of children under 5 years |  | -0.01 | -0.00 |  | 0.02 | 0.02 |
|  |  | (0.06) | (0.05) |  | (0.07) | (0.07) |
| Wealth quintile: Poorer |  | 0.28 | 0.28 |  | 0.18 | 0.17 |
|  |  | (0.11)** | (0.11)*** |  | (0.13) | (0.12) |
| Wealth quintile: Middle |  | 0.31 | 0.30 |  | 0.21 | 0.19 |
|  |  | (0.13)** | (0.12)** |  | (0.16) | (0.15) |
| Wealth quintile: Richer |  | 0.38 | 0.38 |  | 0.41 | 0.40 |
|  |  | (0.13)*** | (0.12)*** |  | (0.16)*** | (0.15)*** |
| Wealth quintile: Richest |  | 0.62 | 0.61 |  | 0.66 | 0.62 |
|  |  | (0.13)*** | (0.13)*** |  | (0.16)*** | (0.15)*** |
| Total land cultivated (in acres) |  | 0.02 | 0.02 |  | 0.02 | 0.02 |
|  |  | (0.02) | (0.02) |  | (0.03) | (0.03) |
| Duration of SHG membership (years) |  | 0.01 | 0.01 |  | 0.00 | 0.00 |
|  |  | (0.01)** | (0.01)* |  | (0.01) | (0.01) |
| Total # group members present for experiment from this group |  | 0.00 | 0.01 |  | 0.01 | 0.01 |
|  |  | (0.01) | (0.01) |  | (0.01) | (0.01) |
| Total #food groups consumed by women |  |  | -0.05 |  |  | -0.04 |
|  |  |  | (0.03) |  |  | (0.04) |
| Currently have a home garden |  |  | 0.11 |  |  | 0.18 |
|  |  |  | (0.08) |  |  | (0.09)** |
| Grows vegetables on farm |  |  | -0.09 |  |  | -0.07 |
|  |  |  | (0.07) |  |  | (0.09) |
| Thinks members should split produce equally |  |  | 0.31 |  |  | 0.34 |
|  |  |  | (0.09)*** |  |  | (0.12)*** |
| Time spent working (in completed hours) |  |  | 0.00 |  |  | -0.01 |
|  |  |  | (0.02) |  |  | (0.02) |
| Perceived group cohesiveness |  |  | 0.04 |  |  | -0.03 |
|  |  |  | (0.06) |  |  | (0.06) |
| Perceived ability of the agent |  |  | 0.26 |  |  | 0.22 |
|  |  |  | (0.04)*** |  |  | (0.05)*** |
| Constant | 7.22 | 7.19 | 5.66 | 7.15 | 7.23 | 6.12 |
|  | (0.09)*** | (0.32)*** | (0.46)*** | (0.07)*** | (0.35)*** | (0.50)*** |
| *R*^2^ | 0.03 | 0.12 | 0.16 | 0.01 | 0.09 | 0.13 |
| *N* | 2,223 | 2,223 | 2,223 | 1,465 | 1,465 | 1,465 |
| Mean of Control | 7.22 | 7.22 | 7.22 | 7.15 | 7.15 | 7.15 |
| Controls |  | Restricted | Full |  | Restricted | Full |
| For columns 1-3 the relevant control group is those SHGs that did not receive information and were matched to a high-ethnic group agent. For columns 4-6 it is those SHGs that were matched to same ethnicity agents and did not receive information. Restricted controls include respondent age, marital status (1/0), ethnicity (=ST), religion (=Hindu), employment status (1/0), education, HH size, number of children under the age of 5 years, wealth quintiles, total land cultivated, number of years they have been an SHG member and the number of SHG members present at the experiment. Full controls additionally include #food groups consumed, have kitchen garden (1/0), grow vegetables on their land (1/0), agree that produce should be split equally (1/0), dummies for group cohesion and for agent ability. Standard errors (in parentheses) clustered at the SHG level. * *p*<0.1 ** *p*<0.05; *** *p*<0.01. | | | | | | |

**Table A2.5: Association of the information and ethnicity treatments with willingness to contribute labor hours (full estimation)**

|  | Full sample | | | Groups matched to same-ethnicity agents | | |
| --- | --- | --- | --- | --- | --- | --- |
| Information treatment | -1.37 | -0.94 | -0.88 | -0.45 | -0.29 | -0.25 |
|  | (1.23) | (1.25) | (1.18) | (0.85) | (0.77) | (0.74) |
| Low ethnic group agent | -4.50 | -3.88 | -3.60 |  |  |  |
|  | (1.16)*** | (1.31)*** | (1.25)*** |  |  |  |
| Information treatment*Low ethnic group agent | 1.70 | 1.08 | 0.79 |  |  |  |
|  | (1.37) | (1.49) | (1.43) |  |  |  |
| Age |  | 0.02 | 0.02 |  | 0.03 | 0.03 |
|  |  | (0.02) | (0.01) |  | (0.02)* | (0.02) |
| Married |  | 1.24 | 0.92 |  | 1.07 | 0.70 |
|  |  | (0.44)*** | (0.41)** |  | (0.52)** | (0.50) |
| Scheduled Tribe |  | -0.53 | -0.62 |  | -3.53 | -3.46 |
|  |  | (0.75) | (0.72) |  | (0.84)*** | (0.79)*** |
| Religion: Hindu |  | -1.13 | -1.01 |  | -0.34 | -0.19 |
|  |  | (0.57)** | (0.56)* |  | (0.54) | (0.56) |
| Respondent woman is employed |  | 0.82 | 0.53 |  | 0.88 | 0.64 |
|  |  | (0.39)** | (0.39) |  | (0.48)* | (0.48) |
| Completed less than grade 5 |  | 0.56 | 0.30 |  | 0.86 | 0.62 |
|  |  | (0.43) | (0.43) |  | (0.50)* | (0.50) |
| Completed between grades 5 and 8 |  | 1.04 | 0.86 |  | 1.88 | 1.80 |
|  |  | (0.64) | (0.65) |  | (0.83)** | (0.85)** |
| Completed grade 9 or higher |  | 1.71 | 1.21 |  | 2.30 | 1.71 |
|  |  | (0.70)** | (0.71)* |  | (0.92)** | (0.94)* |
| Household size |  | -0.11 | -0.09 |  | -0.08 | -0.07 |
|  |  | (0.10) | (0.10) |  | (0.12) | (0.12) |
| # of children under 5 years |  | -0.07 | -0.13 |  | -0.17 | -0.26 |
|  |  | (0.27) | (0.26) |  | (0.34) | (0.34) |
| Wealth quintile: Poorer |  | 0.35 | -0.03 |  | 0.44 | -0.10 |
|  |  | (0.45) | (0.45) |  | (0.56) | (0.58) |
| Wealth quintile: Middle |  | 1.20 | 0.54 |  | 1.41 | 0.64 |
|  |  | (0.47)** | (0.49) |  | (0.57)** | (0.62) |
| Wealth quintile: Richer |  | 0.91 | 0.24 |  | 1.33 | 0.55 |
|  |  | (0.50)* | (0.49) |  | (0.59)** | (0.61) |
| Wealth quintile: Richest |  | 0.14 | -0.65 |  | 0.21 | -0.62 |
|  |  | (0.63) | (0.66) |  | (0.79) | (0.86) |
| Total land cultivated (in acres) |  | 0.09 | -0.01 |  | 0.10 | 0.04 |
|  |  | (0.13) | (0.11) |  | (0.15) | (0.14) |
| Duration of SHG membership (years) |  | 0.04 | 0.02 |  | 0.03 | 0.01 |
|  |  | (0.05) | (0.05) |  | (0.07) | (0.06) |
| Total #group members present for experiment |  | 0.04 | 0.03 |  | -0.03 | -0.02 |
|  |  | (0.15) | (0.14) |  | (0.11) | (0.11) |
| Total #food groups consumed by women |  |  | 0.41 |  |  | 0.40 |
|  |  |  | (0.16)** |  |  | (0.19)** |
| Currently have a home garden |  |  | 1.18 |  |  | 1.28 |
|  |  |  | (0.37)*** |  |  | (0.46)*** |
| Grows vegetables on farm |  |  | 0.29 |  |  | 0.03 |
|  |  |  | (0.42) |  |  | (0.55) |
| Thinks members should split produce equally |  |  | -1.08 |  |  | -1.33 |
|  |  |  | (0.33)*** |  |  | (0.44)*** |
| Time spent working (in completed hours) |  |  | 0.23 |  |  | 0.16 |
|  |  |  | (0.06)*** |  |  | (0.07)** |
| Perceived group cohesiveness |  |  | 0.73 |  |  | 0.93 |
|  |  |  | (0.30)** |  |  | (0.37)** |
| Perceived ability of the agent |  |  | 0.03 |  |  | -0.01 |
|  |  |  | (0.13) |  |  | (0.18) |
| Constant | 10.79 | 10.69 | 6.63 | 6.73 | 9.38 | 6.42 |
|  | (1.32)*** | (2.29)*** | (2.37)*** | (0.75)*** | (2.47)*** | (2.66)** |
| *R*^2^ | 0.06 | 0.08 | 0.11 | 0.00 | 0.10 | 0.14 |
| *N* | 2,223 | 2,223 | 2,223 | 1,465 | 1,465 | 1,465 |
| Mean of Control | 11.76 | 11.76 | 11.76 | 9.62 | 9.62 | 9.62 |
| Controls |  | Restricted | Full |  | Restricted | Full |
| For columns 1-3 the relevant control group is those SHGs that did not receive information and were matched to a high-ethnic group agent. For columns 4-6 it is those SHGs that were matched to same ethnicity agents and did not receive information. Restricted controls include respondent age, marital status (1/0), ethnicity (=ST), religion (=Hindu), employment status (1/0), education, HH size, number of children under the age of 5 years, wealth quintiles, total land cultivated, number of years they have been an SHG member and the number of SHG members present at the experiment. Full controls additionally include #food groups consumed, have kitchen garden (1/0), grow vegetables on their land (1/0), agree that produce should be split equally (1/0), dummies for group cohesion and for agent ability. Standard errors (in parentheses) clustered at the SHG level. * *p*<0.1 ** *p*<0.05; *** *p*<0.01. | | | | | | |

# Appendix A3: Study site and scoping

Our study was conducted in Baghmundi community development block in Purulia district of West Bengal. At 20%, West Bengal’s rate of poverty is close to the national average of 22%. However, there is considerable heterogeneity across the state and Purulia is among the districts with the highest rate of poverty in the state, with an estimated 31-38% of the population living below the poverty line. While the district is largely rural, with few small urban centers, Baghmundi block is entirely rural, and predominantly agriculture depend. OBCs and STs make up 61% and 24% of the population of Baghmundi respectively, making them the two largest ethnic groups in the block.

As part of exploratory research, we conducted extensive scoping visits and discussions with PRADAN to inform the design of key elements of the experiment, including site selection, design of information treatment and recruiting agents. As part of this visit, we met with SHGs of different ethnic groups including mixed groups with members from different ethnic communities. We found that women from high ethnicity groups were financially secure and unwilling to contribute labor hours to the cultivation of foods they could easily purchase in the market. However, there was widespread willingness among the members of other ethnic groups to contribute to a group-owned kitchen garden. SHGs of mixed ethnic composition were not as cohesive or cooperative as those in which all members belonged to the same ethnicity. In mixed groups we found disagreement between women from different communities on how to share labor hours and output. This stemmed particularly from the differing opportunity cost of time, as women from SC groups worked predominantly as wage laborers on farms and would thus forego wages to work on communal farms. These women wanted output from communal farms to be shared in proportion to time contributed, whereas women from other communities (OBC and high ethnic/caste groups), who largely worked on their own farms were in favor of sharing output equally.

Tribal populations (STs) in this area were distinctly worse off on almost all measurable welfare indicators, and there was broad recognition that they were the lowest rung in the caste/ethnicity hierarchy. They were also largely geographically isolated, being confined to a part of the block (Ajodhya hills) that is remote and relatively inaccessible. Someone familiar with the context would be able to distinguish members of different ethnic groups from their appearance, but even if not, individual names were a clear indication of ethnic group identity.

Large scale data corroborates the findings of our scoping visits, and the marginalization of STs relative to other ethnic groups is evident in their socioeconomic and demographic characteristics. Using the fourth round of the National Family Health Survey (NFHS)-4 (2015-16) we compared caste and tribe groups in West Bengal across a small set of indicators (Appendix Table A.1). ST women reported the lowest education among all ethnic groups, had lower women’s body mass index on average, and belonged to households that predominantly came from the poorest two wealth quintiles, as calculated using state-specific rural population wealth indices.

It is to be noted that while sub-castes or *jatis* exist within the larger ST and OBC caste groups, SHGs tend to be homogenous even on sub-caste. Moreover, within STs, sub-castes are largely linear in hierarchy. Finally, among OBCs and ST’s, land is abundant and, if no member was willing to loan household land for the community garden, it could be rented at a very nominal price, making kitchen gardens feasible to implement.

Our aim in recruiting agents was to minimize variability in quality of information delivery and other soft skills such as confidence and familiarity with material. To do this, we worked with PRADAN to recruit agents who had experience with community engagement as part of other routine outreach and activities. The content of the information treatment and control was co-created by the researchers and PRADAN, and care was taken to center the content in local food preferences, availability and feasibility.

# Appendix A4: Nutrition knowledge test

| Q1. Which of these types of foods are energy giving foods, i.e. give you the energy you need to run and play and do work? | 1. **Oil, ghee, sugar, potato and grains** 2. Green leafy vegetables and Fruits 3. Don’t know |
| --- | --- |
| Q2. Which of these types of foods are body building foods, i.e. help build bones and tissue? | 1. Green leafy vegetables and fruits 2. **All types of pulses, animal source foods including milk and milk products** 3. Don’t know |
| Q3. Which of these types of foods are protective foods, i.e. prevent you from falling sick? | 1. **Green leafy vegetables and Fruits** 2. Oil, ghee, sugar, potato and grains 3. Don’t know |
| Q4. Which of the following foods is rich in iron? | 1. Oil, ghee 2. **Dark green vegetables** 3. Don’t know |
| Q5. How should a pregnant/lactating woman eat in comparison with a non-pregnant woman to provide good nutrition to her baby and help the baby grow? | 1. Eat same at each meal 2. **Eat more at each meal (eat more food each day)** 3. Don’t know |
| Q6. Which of the following constitutes a tri-coloured meal? | 1. **Lentils, rice/chapatti and spinach** 2. Curd, rice/chapatti and potato 3. Don’t know |
| Q7. Which of these vegetables can be grown in kitchen garden in the summer/kharif? | 1. **Bottle guard (Lauki)** 2. Cauliflower (Gobhi) 3. Don’t know |
| Q8. Which of the following food is rich in vitamin A? | 1. **Carrot** 2. Brinjal (Baingan) 3. Don’t know |
| Q9. How can anemia be prevented? | 1. Drinking clean water 2. **Eat iron-rich food like green, leafy vegetables** 3. Don’t know |
| Q10. What causes anemia? | 1. **Lack of iron in the diet /not eating iron rich food** 2. Drinking dirty water 3. Don’t know |
